# Perilipin2-dependent lipid droplets accumulation promotes metastasis of oral squamous cell carcinoma via epithelial-mesenchymal transition

**DOI:** 10.1038/s41420-025-02314-1

**Published:** 2025-01-28

**Authors:** Jiayu Zhang, Jianmin Peng, Siyu Wang, Li Wang, Yutong Sun, Juan Xia, Bin Cheng, Qinchao Hu

**Affiliations:** 1 Hospital of Stomatology, Sun Yat-Sen University, Guangzhou, China; 2 Guangdong Provincial Key Laboratory of Stomatology, Guanghua School of Stomatology, Sun Yat-Sen University, Guangzhou, China

**Keywords:** Oral cancer, Epithelial-mesenchymal transition

## Abstract

Emerging evidence shows that lipid metabolic reprogramming plays a vital role in tumor metastasis. The effect and mechanism of fatty acids and lipid droplets (LDs), the core products of lipid metabolism, on the metastasis of oral squamous cell carcinoma (OSCC), need further exploration. In this study, the influence of palmitic acid (PA) and oleic acid (OA) on the migration and invasion ability of OSCC cells was determined by in vitro experiments. Genetic manipulation of PLIN2 was performed to explore its effect on the accumulation of LDs and OSCC metastasis. Possible mechanisms of these biological effects were clarified by detecting the levels of epithelial-mesenchymal transition (EMT) markers and phosphatidylinositol 3-kinase (PI3K) pathway proteins as well as conducting various bioinformatics analyses. The results indicated that PA/OA promoted the migration and invasion of OSCC cells and induced PLIN2-dependent LDs accumulation in vitro. Knockdown of PLIN2 inhibited the LDs accumulation and the migration and invasion of OSCC cells in vitro, while overexpression of PLIN2 enhanced those of OSCC cells in vitro and also promoted the metastasis of OSCC in vivo. Besides, PLIN2 up-regulation activated the PI3K pathway and subsequently enhanced EMT in OSCC cells in vitro. OSCC patients with higher PLIN2 expression possessed poorer prognosis and higher sensitivity to chemotherapy drugs (1S,3 R)-RSL3 and ML-210. In conclusion, PLIN2-dependent LDs accumulation could promote the metastasis of OSCC cells by regulating EMT. PLIN2 might be a potential therapeutic target for OSCC patients, especially those with obesity.

## Introduction

Oral squamous cell carcinoma (OSCC) is an invasive epithelial tumor accounting for about 90% of oral malignancies, with a 5-year survival rate of around 60% [1]. Tumor metastasis is a complex cascade, making it the primary cause of cancer-related mortality [2]. With the increasing proportion of obese people worldwide, obesity-induced lipid metabolism abnormalities are extensively studied as risk factors for diverse tumor metastasis [3, 4], including breast cancer [5–7], ovarian cancer [8], colon cancer [9], prostate cancer [10], thyroid cancer [11], and so on. We previously found that obesity was an important risk factor for OSCC [12] and could further promote tumor metastasis and recurrence in early OSCC patients [13], and some lipid metabolism related genes might serve as biomarkers to predict patients’ prognosis [14, 15]. Nevertheless, more specific mechanisms by which obesity and lipid metabolism regulate the progression of OSCC remain to be further clarified to provide potential therapeutic targets.

Obese patients have higher levels of free fatty acids (FAs) in their plasma [16]. Lipid metabolic reprogramming presents as a complex network, with FAs cycling making the core node of this framework [4]. FAs are essential components of cell membranes and can partake in signal transduction and energy storage as well [17]. Tumor cells can enhance the de novo lipogenesis to meet the increased metabolic requirements [18], while recent studies gradually indicated that exogenous FAs might offer a selective advantage for fueling cancer metastasis [19, 20]. Palmitic acid (PA) and oleic acid (OA) are typical representatives of saturated fatty acids and monounsaturated fatty acids, respectively, and both account for a high proportion of plasma free FAs. However, the exact influences of PA and OA on cancer development are still controversial [16, 21–23]. Consequently, untangling the specific role of PA and OA in the metastasis of OSCC might provide new insights into the behavior and diet advice of OSCC patients. Knowledge of the mechanisms by which FAs regulate specific gene expression may identify important risk factors and therapeutic targets for OSCC.

Fatty acids can be esterified into triglycerides through various enzymatic reactions, which can be encapsulated in lipid droplets (LDs) to prevent excess fatty acids and their derivatives from producing cytotoxicity. As dynamic organelles for lipid storage, LDs also play important roles in lipid metabolism. LDs are made up of a hydrophobic core of triacylglycerol and cholesteryl esters surrounded by a monolayer of phospholipids [24]. LDs accumulation in non-adipose tissues has been considered a new hallmark of cancer, accompanied by some LDs-related proteins such as the perilipin family. Other than simply participating in lipid storage, the perception of the perilipin 2 (PLIN2) role has been expanded to a series of metabolic diseases, neurodegenerative diseases, and cancers [25–31]. However, the specific picture that elucidates the roles of LDs and PLIN2 in OSCC is still missing currently.

Epithelial-mesenchymal transition (EMT) mainly functions during the priming phase of the distant metastasis and could be induced by LDs accumulation in specific tumors [32, 33]. EMT was commonly seen in some tumor cells and making the cells possess the ability of invasion via losing epithelial traits and demonstrating mesenchymal cell characteristics [34, 35]. Supplementation with exogenous PA or OA could promote tumor cell migration and expression of EMT markers in various cancers, such as hepatocellular carcinoma [36, 37]. Uncovering the detailed mechanisms would provide a rationale for precision therapy aiming at the molecular targets.

Here, we demonstrated that both PA and OA promoted the migratory and invasive abilities of OSCC cells. To be specific, PA induced PLIN2-dependent LDs accumulation, thereby up-regulating the emergence of EMT in OSCC cells. Furthermore, PLIN2 was positively related to higher metastasis and poorer prognosis in OSCC patients. These results suggested that PLIN2 might presumably be a potential molecular biomarker and therapeutic target for OSCC patients, especially those with obesity.

## Results

### FAs stimulation promotes the migration and invasion of OSCC cells

**Fig. 1:**
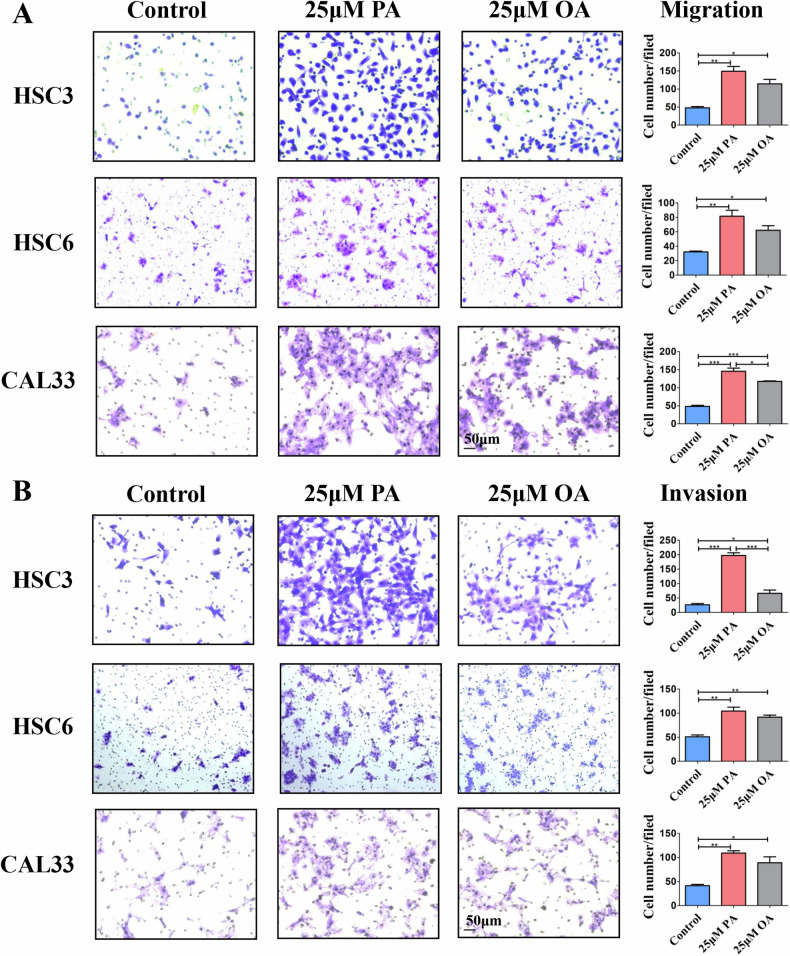
FAs stimulation promotes the migration and invasion of OSCC cells. Transwell results showed that 25 μM PA or OA stimulation increased the in vitro migration (**A**) and invasion (**B**) ability of different OSCC cells. Bar: 50 μm. *P < 0.05, **P < 0.01, ***P < 0.001. FAs Fatty acids, PA Palmitic acid, OA Oleic acid.

HSC3/HSC6/CAL33 cells were treated with the indicated concentrations (12.5, 25, 50 μM) of PA or OA in vitro for 24 h and their migratory abilities were detected via the wound healing assay. The results showed that both PA (supplementary Fig. 1) and OA (supplementary Fig. 2) presented the most significant promoting effect on the migration of OSCC cells at 25 μM. Further transwell assays verified that 25 μM PA/OA effectively enhanced the migration and invasion of OSCC cells (Fig. 1). We repeated the above transwell assay using 3 other OSCC cell lines and obtained consistent results (supplementary Fig. 3A, B). Compared with OA, the promoting effect of PA was more powerful. Besides, the CCK-8 assay revealed that both FAs had no significant influence on cell viability at 25 μM (supplementary Fig. 3C), while significantly reduced the cell viability at 50 μM (supplementary Fig. 3D), indicating that high concentration of FAs may be lipotoxic, which attenuates the effect of FAs in promoting cell migration/invasion.

### FAs stimulation induces PLIN2-dependent LDs accumulation in OSCC cells

**Fig. 2:**
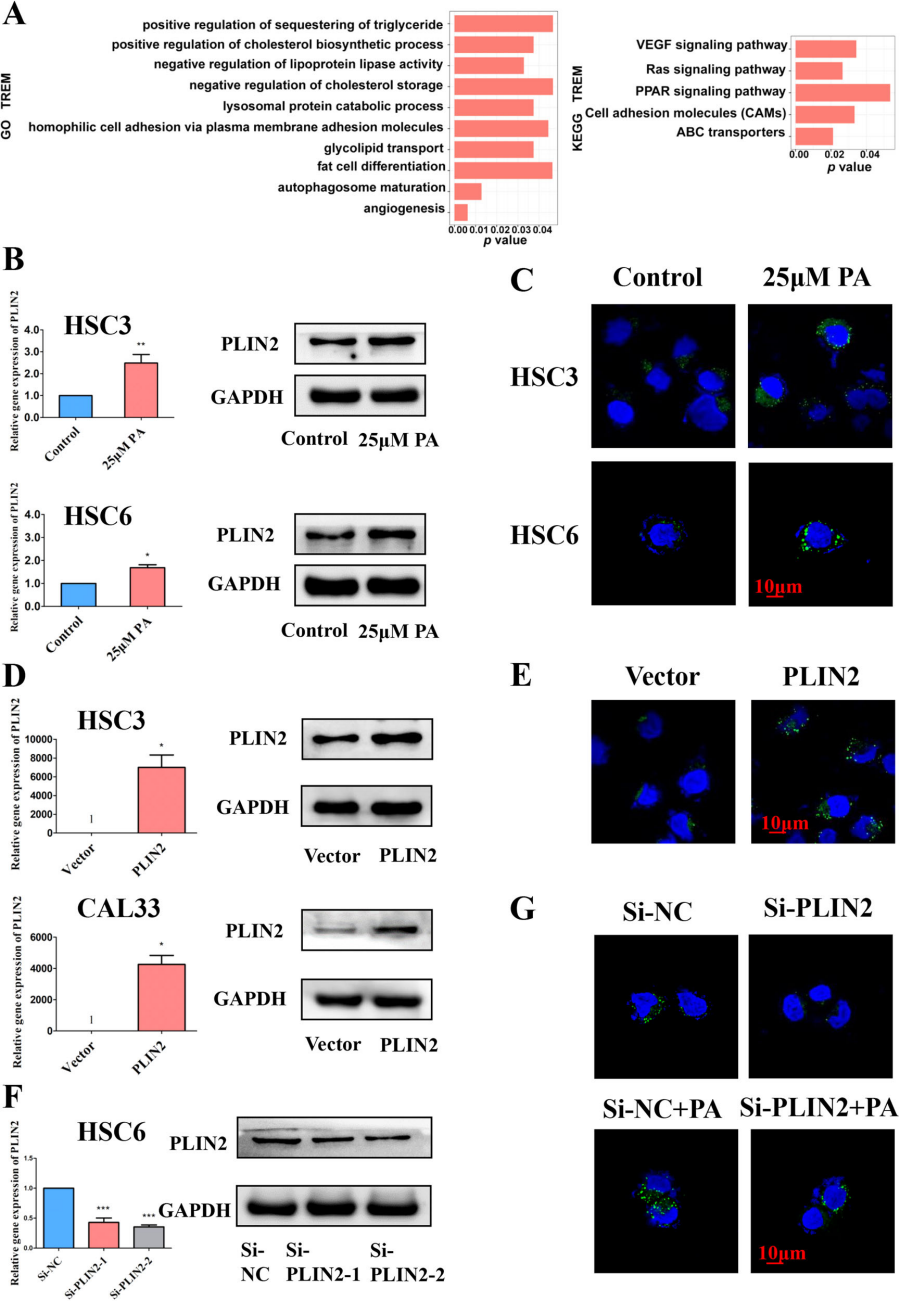
FAs stimulation induces PLIN2-dependent LDs accumulation in OSCC cells. **A** GO and KEGG analysis showed that the differentially expressed genes after PA stimulation were enriched in diverse aspects closely related to tumor metastasis like lipid metabolism, adhesion, and angiogenesis. **B** qRT-PCR and WB results showed that the expression level of PLIN2 in both HSC3 and HSC6 cells was up-regulated after 25 μM PA stimulation. **C** BODIPY staining results showed that 25 μM PA stimulation enhanced LDs accumulation in different OSCC cells. **D** qRT-PCR and WB results confirmed that the expression levels of PLIN2 in HSC3 and CAL33 cells were significantly increased after stable infection with lentiviral vectors. **E** BODIPY staining results showed that overexpression of PLIN2 enhanced LDs accumulation in HSC3 cells. **F** qRT-PCR and WB results confirmed that the expression level of PLIN2 in HSC6 cells was significantly decreased after transient transfection with Si-RNA. **G** BODIPY staining results showed that knockdown of PLIN2 reduced the accumulation of LDs in HSC6 cells, and the promoting effect of 25 μM PA stimulation on intracellular LDs accumulation was also decreased correspondingly. Bar: 10 μm. The green fluorescence in (**C**, **E**, **G**) is the BODIPY staining of LDs, and the blue fluorescence is the Hoechst staining of the nucleus. *P < 0.05, **P < 0.01, ***P < 0.001. FAs Fatty acids, PA Palmitic acid, LDs Lipid droplets, NC Negative control.

To investigate the underlying mechanism, HSC3 cells stimulated with 25 μM PA were collected for mRNA sequencing. The same batch of samples was used for the transwell migration assay confirming the effectiveness of PA treatment (supplementary Fig. 4A). In comparison with the negative control sample, 148 DEGs were screened out with 71 up-regulated and 77 down-regulated (supplementary Fig. 4B and supplementary Table 1). For GO and KEGG analysis, the DEGs were enriched in lipid metabolism, biological adhesion, and movement, which were closely related to tumor metastasis (Fig. 2A).

Among all the DEGs, PLIN2 drew our attention resulting from its various functions in LDs biogenesis, metabolic diseases, neurological degenerative diseases, and cancers [25–31]. Within our sequencing results, the expression of PLIN2 in OSCC cells after PA stimulation was significantly upregulated (supplementary Fig. 4C). Therefore, we hypothesized that the role of PA in promoting migration and invasion of OSCC cells may be related to the upregulation of PLIN2 and the associated formation of LDs. To verify the above hypothesis, we detected the background expression level of PLIN2 in different OSCC cell lines (supplementary Fig. 4D) and the corresponding change after PA stimulation by PCR and WB. The results confirmed the upregulation of PLIN2 in OSCC cells treated with PA (Fig. 2B). Consistently, LDs accumulation also increased after PA stimulation (Fig. 2C). To further interfere with the PLIN2 expression, HSC3 and CAL33 cells were transfected with lentivirus to construct cell lines stably overexpressing PLIN2 while HSC6 cells were transfected with Si-RNA targeting PLIN2 to transiently knockdown its expression. After confirming the efficiency of overexpression and knockdown with PCR and WB, BODIPY staining was conducted and further demonstrated the necessary role of PLIN2 during LDs formation (Fig. 2D–G). These results implied that PA could increase the PLIN2 expression, which dependently promoted the LDs accumulation.

### PLIN2-dependent LDs accumulation increases the migration and invasion of OSCC cells

**Fig. 3:**
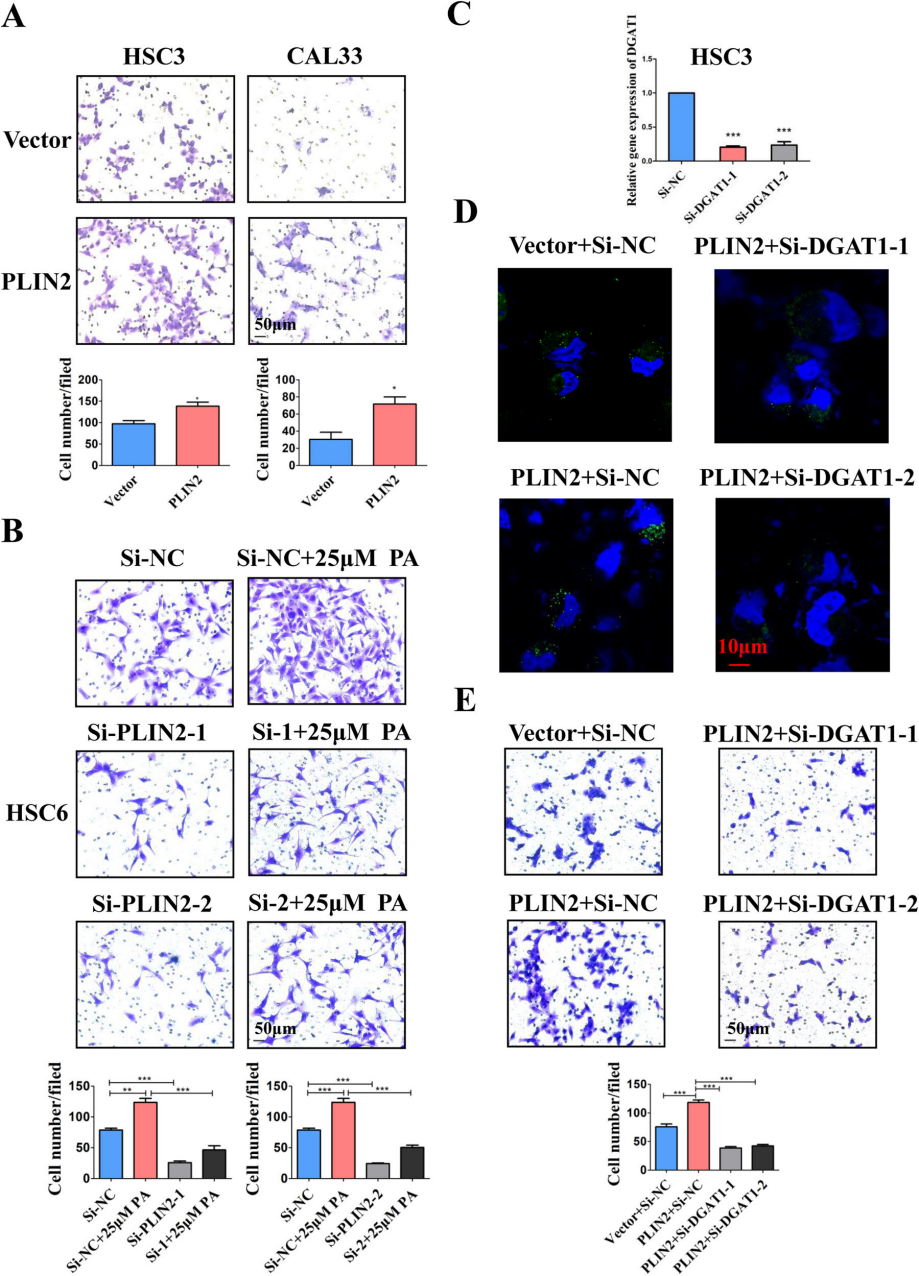
PLIN2 can promote the invasion of OSCC cells via the accumulation of LDs. **A** Transwell results showed that overexpression of PLIN2 can increase the in vitro invasion ability of HSC3 and CAL33 cells. Bar: 50 μm. **B** Transwell results showed that knockdown of PLIN2 decreased the promoting effect of 25 μM PA stimulation on the in vitro invasion of HSC6 cells. Bar: 50 μm. **C** qRT-PCR results confirmed that after transient transfection with Si-RNA, the expression level of DGAT1 in HSC3 cells stably overexpressing PLIN2 was significantly reduced. **D** BODIPY staining results showed that knockdown of DGAT1 reduced LDs generation in HSC3 cells stably overexpressing PLIN2. Bar: 10 μm. The green fluorescence in this figure is the BODIPY staining of LDs, and the blue fluorescence is the Hoechst staining of the nucleus. **E** Transwell results indicated that knockdown of DGAT1 significantly decreased the in vitro invasion ability of HSC3 cells stably overexpressing PLIN2. Bar: 50 μm. *P < 0.05, **P < 0.01, ***P < 0.001. PA: Palmitic acid; NC: Negative control; LDs: Lipid droplets.

Transwell assays further clarified that the trends of PLIN2-dependent LDs accumulation were consistent with the change in biological behaviors of OSCC cells. Overexpression of PLIN2 significantly enhanced the invasion and migration of OSCC cells (Fig. 3A and supplementary Fig. 5A) while knockdown of PLIN2 inhibited that of OSCC cells (Fig. 3B and supplementary Fig. 5B). Besides, unlike when PLIN2 was normally expressed, PA could not significantly promote the invasion of OSCC cells if PLIN2 was knockdown (Fig. 3B). CCK-8 assay suggested that PLIN2 overexpression had no significant effect on OSCC cell proliferation (supplementary Fig. 5C).

LDs accumulation required normal expression of other LDs-related enzymes to ensure the biogenesis of LDs. Knockdown of DGAT1, a rate-limited enzyme in the process of triglyceride synthesis, would significantly inhibit the LDs formation of OSCC cells overexpressed with PLIN2 (Fig. 3C, D). The corresponding invasive ability (Fig. 3E) and migratory ability (supplementary Fig. 5D) were decreased in the meanwhile. These results emphasized that successful synthesis of LDs was a prerequisite for PLIN2 overexpression exerting the promoting effect on the migration and invasion of OSCC cells.

### PLIN2-dependent LDs accumulation enhances EMT in OSCC cells via activating the PI3K/AKT/mTOR pathway

**Fig. 4:**
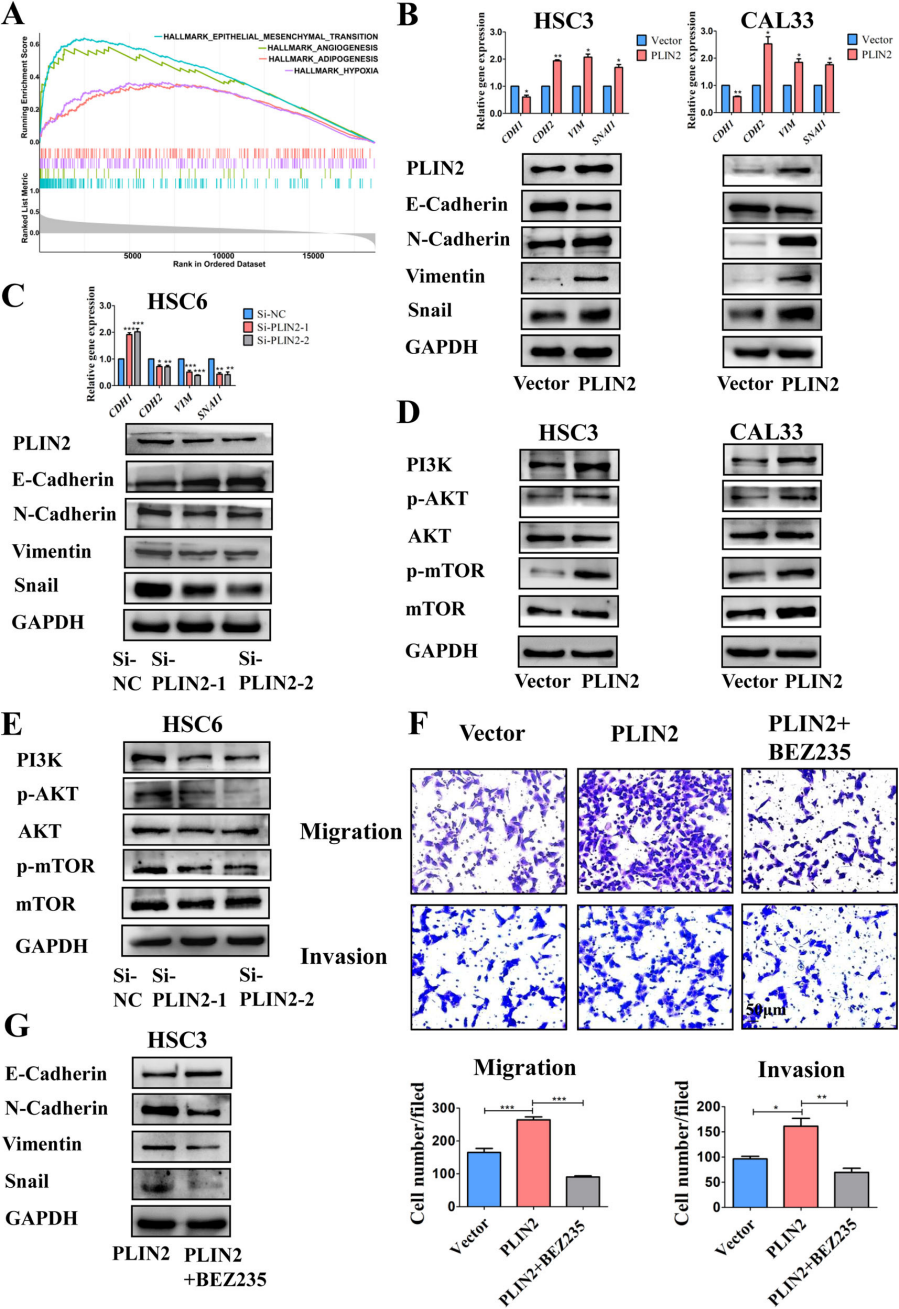
PLIN2 promoted the EMT, migration and invasion of OSCC cells by activating the PI3K/AKT/mTOR pathway. **A** The results of GSEA suggested that genes closely related to PLIN2 in the TCGA database were enriched in the classical pathways highly relevant to tumor metastasis, such as EMT, angiogenesis, adipogenesis, and hypoxia. **B**, **C** qRT-PCR and WB results showed that overexpression of PLIN2 was accompanied by the decreased expression of epithelial cell marker E-cadherin (encoding gene: *CDH1*), while the expression of mesenchymal cell markers N-cadherin (encoding gene: *CDH2*), Vimentin (encoding gene: *VIM*), and transcription factor Snail (encoding gene: *SNAI1*) was increased. Knockdown of PLIN2 showed converse trends, which was consistent with the above results. **D**, **E** WB results demonstrated that after the overexpression of PLIN2, the expression levels of PI3K protein and its downstream phosphorylated proteins p-AKT and p-mTOR were increased. Knockdown of PLIN2 showed converse trends, which was consistent with the above results. **F** Transwell results indicated that treatment with 15 nM PI3K pathway inhibitor BEZ235 for 24 h significantly inhibited the in vitro migration and invasion abilities of HSC3 cells stably overexpressing PLIN2. **G** WB results verified that treatment of HSC3 cells stably overexpressing PLIN2 with 15 nM BEZ235 for 24 h resulted in the up-regulation of E-cadherin and the down-regulation of N-cadherin, Vimentin, and Snail in cells. Bar: 50 μm. *P < 0.05, **P < 0.01, ***P < 0.001. GSEA: Gene Set Enrichment Analysis; EMT: Epithelial-mesenchymal transition; NC Negative control.

GSEA results indicated that PLIN2-related genes in the TCGA-OSCC dataset were enriched in the pathways closely relevant to tumor metastasis like EMT, angiogenesis, adipogenesis, hypoxia, and so on (Fig. 4A). Considering the well-accepted role of EMT in tumor metastasis, we detected the expression of EMT-related markers in OSCC cells after overexpression/knockdown of PLIN2 by RT-PCR and WB. We found that overexpression of PLIN2 could result in the decreased expression of epithelial cell marker E-cadherin (encoding gene: *CDH1*), while the expression of mesenchymal cell markers N-cadherin (encoding gene: *CDH2*), Vimentin (encoding gene: *VIM*), and transcription factor Snail (encoding gene: *SNAI1*) was increased (Fig. 4B). Consistently, knockdown of PLIN2 upregulated the expression of epithelial cell marker and downregulated the mesenchymal cell markers (Fig. 4C). These results indicated that the role of PLIN2 in promoting migration and invasion might be related to EMT.

The PI3K/AKT/mTOR pathway plays a crucial role in EMT. Our data showed that overexpression of PLIN2 upregulated the expression of PI3K, thereby activating its downstream target proteins p-Akt and p-mTOR (Fig. 4D). Correspondingly, knockdown of PLIN2 inhibited this pathway (Fig. 4E). We further treated the cells with PI3K/mTOR dual inhibitor BEZ235. It turned out that BEZ235 significantly reduced the migration and invasion of OSCC cells overexpressing PLIN2 (Fig. 4F). The corresponding expression of E-cadherin was increased and that of N-cadherin, Vimentin, and Snail was decreased (Fig. 4G). These results suggested that the PLIN2-dependent LDs accumulation might induce EMT of OSCC cells via activation of the PI3K/AKT/mTOR pathway, thus enhancing their migratory and invasive abilities.

### Upregulation of PLIN2 accelerates in vivo metastasis of OSCC cells and predicts poor prognosis of OSCC patients

**Fig. 5:**
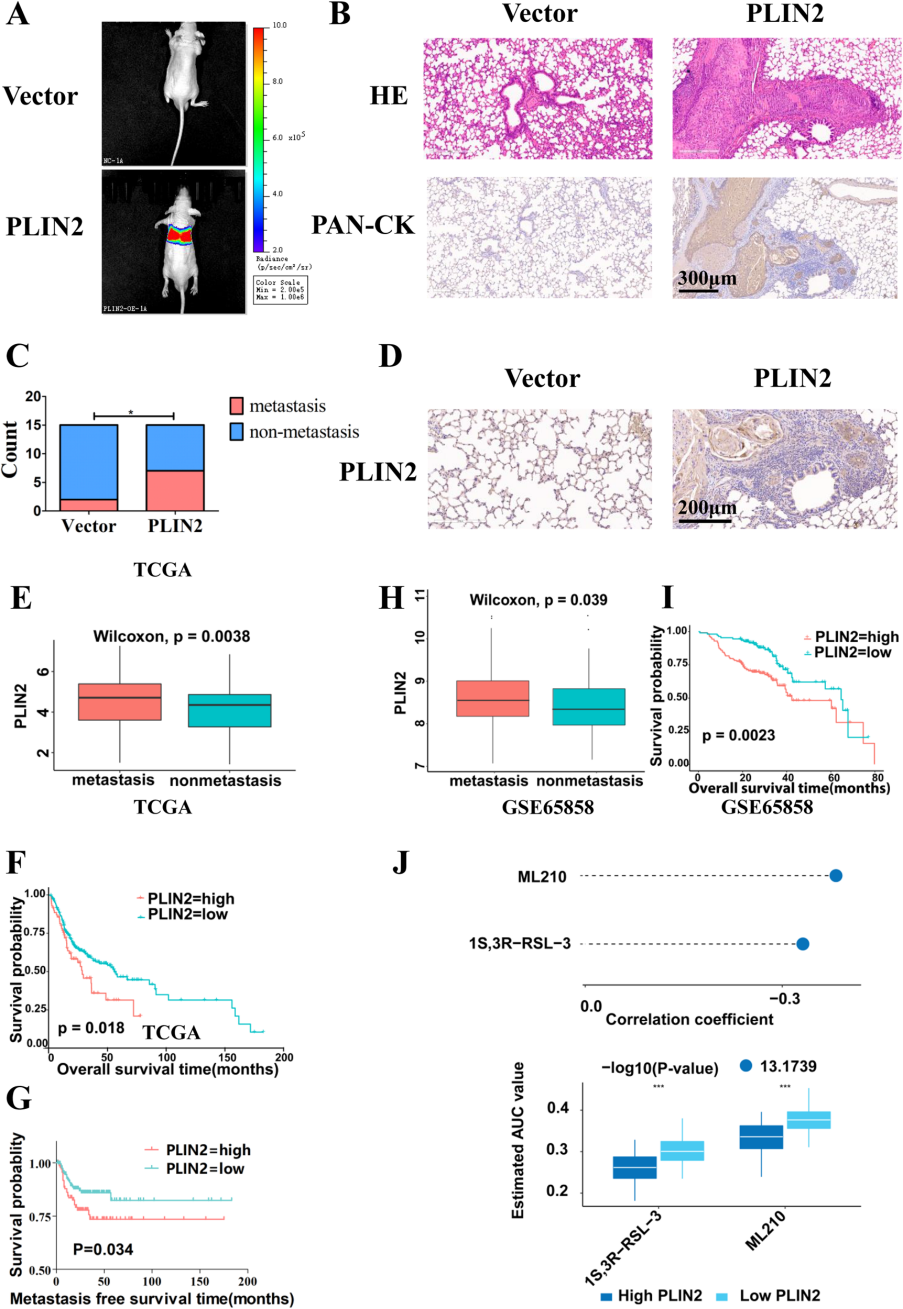
Upregulation of PLIN2 accelerates in vivo metastasis of OSCC cells and predicts poor prognosis of OSCC patients. **A** In vivo imaging results showed that HSC3 cells stably overexpressing PLIN2 injected into the nude mice through the tail vein could form tumor metastases in the lungs. **B** HE staining results presented significant tumor metastases in the lungs of nude mice injected with HSC3 cells stably overexpressing PLIN2 and the corresponding PAN-CK staining confirmed that the lung metastases were of epithelial origin. Bar: 300 μm. **C** The lung metastasis rate of nude mice in the experimental group was significantly higher than that in the control group (*n* = 15). **D** IHC staining of PLIN2 showed that high expression of PLIN2 was detected in tumor metastasis sites in lung tissues. Bar: 200 μm. **E** Analysis of TCGA database showed that PLIN2 was highly expressed in OSCC patients with lymph node metastasis. **F**, **G** Analysis of TCGA database showed that OSCC patients with high PLIN2 expression possessed shorter overall survival time and metastasis-free survival time than those with low PLIN2 expression. **H** Analysis of the GSE65858 dataset showed that PLIN2 was highly expressed in OSCC patients with lymph node metastasis. **I** Analysis of the GSE65858 dataset showed that OSCC patients with high PLIN2 expression possessed a shorter overall survival time than those with low PLIN2 expression. **J** Comprehensive analysis of TCGA and GDSC databases revealed that tumor chemotherapy drugs (1S,3 R) -RSL3 and ML-210 had higher sensitivity in OSCC patients with high PLIN2 expression. *P < 0.05. GDSC: Genomics of Drug Sensitivity in Cancer.

To further verify the in vitro results presented above, HSC3 cells stably overexpressed with PLIN2 or negative control cells were injected into nude mice via the tail vein (*n* = 15). Tissue HE staining showed that 7 mice in the overexpression group emerged lung metastasis while 2 in the control group, indicating a significant difference in the metastasis rate between the two groups. Besides, the corresponding PAN-CK staining confirmed that the pulmonary metastasis was of epithelial origin (Fig. 5A–C). In addition, the results of PLIN2 staining showed that high expression of PLIN2 was detected in tumor metastasis sites in lung tissues (Fig. 5D). These results suggested that overexpression of PLIN2 could promote the metastasis of OSCC cells in vivo.

Bioinformatic analysis of TCGA and GSE65858 datasets demonstrated that the expression of PLIN2 was significantly higher in OSCC patients with lymph node metastasis (*P* < 0.05) (Fig. 5E, H). In addition, the overall survival and metastasis free survival were significantly poorer in OSCC patients with high PLIN2 expression (*P* < 0.05) (Fig. 5F, G, I), indicating that PLIN2 might promote tumor progression and predict poor prognosis in OSCC patients. Furthermore, we explored the chemotherapy drugs that were sensitive to patients with high PLIN2 expression in the GDSC database. Chemotherapy drugs (1S,3 R)-RSL3 and ML-210 showed higher sensitivity in OSCC patients with high PLIN2 expression, both of which were inhibitors of glutathione peroxidase 4 (GPX4) (Fig. 5J).

## Discussions

The results reported here demonstrated the promoting effect of exogenous FAs on the migration and invasion of OSCC cells. Specifically, exogenous FAs supplement induced the PLIN2-dependent LDs accumulation and activated the PI3K/AKT/mTOR pathway, which promoted the occurrence of EMT and ultimately presented as enhanced metastasis of OSCC cells.

The incidence of obesity is increasing mainly due to the diffusion of industrial food rich in sugar and fat, along with the reduction of physical activity. The known association between obesity and cancer mortality clearly reinforces interest in this research area. Previous studies suggested chronic inflammation and oxidative stress as the underlying links between obesity and cancer, providing a suitable soil for the initiation and progression of neoplasia [38]. A systematic review also implied that this cancer-enhancing crosstalk might be mediated by interleukin-6 (IL-6), tumor necrosis factor-alpha (TNF-alpha), vascular endothelial growth factor (VEGF), and other potential mechanisms waiting for exploration [39]. Other than inflammation, pieces of literature related to lipid metabolism are gradually arising, unveiling the sophisticated lipid regulatory network within tumors on multiple functional levels [40].

Metastatic disease is responsible for more than 90% of all cancer-related deaths, due to the difficulties in the surgical resection or the severe side effects resulting from multiple rounds of chemotherapy and radiation therapy [2]. Metastasis is a complex process that requires sufficient energy and biomass components. Consequently, reprogramming of metabolic programs like aerobic glycolysis and increased glutamine metabolism is necessary for cancer cells. Under energy-deficient conditions, lipid metabolism plays a vital role in the invasion, circulation, and proliferation of cancer cells during metastasis such as energy storage, membranes formation, production of signaling molecules as well as an effective adenosine-triphosphate (ATP) generator via FAs oxidation [4]. The avidity for lipids can be sourced from endogenous synthesis or exogenous supplementation [41]. PA is the principal constituent of refined palm oil with controversial studies arguing its potentially unhealthy effect [42]. Recent research demonstrated that CD36 could mediate PA-induced metastasis of gastric cancer and initiate the metastasis of OSCC with the stimulation of exogenous PA [21, 43]. OA takes up a considerable proportion of FAs in the grease of animals and vegetables. On the one hand, OA-induced recombinant angiopoietin like protein 4 (ANGPTL4) could promote the anoikis resistance and metastasis of head and neck squamous cell carcinoma through upregulating fibronectin [16]. On the other hand, OA could be applied in the treatment of tongue squamous cell carcinomas by inducing apoptosis and autophagy [23]. Our data showed the promotive role of exogenous PA and OA within proper concentration during the migration and invasion of OSCC cells. Hence, OSCC patients could benefit from stricter control of PA and OA uptake in their daily diets.

To prevent lipotoxicity, cells can esterify extra FAs and cholesterol and store them as neutral molecules like triglycerides and sterol ester in LDs, making LDs an important transfer station in lipid metabolism. Accumulation of LDs is now well accepted as a poor prognostic marker of several cancers from both basic research and clinical practice [44, 45]. PLIN2, also known as adipose differentiation-related protein, has been well-established as an LDs coat protein partaking in the storage of lipids. Besides, recent studies implied that the expression of PLIN2 changes in a variety of pathological conditions including cancer [25]. To be specific, overexpression of PLIN2 correlated with a markedly worse prognosis in breast cancer, colon adenocarcinoma, gastric carcinoma, clear cell renal cell carcinoma, and lung adenocarcinoma. The underlying mechanisms by which PLIN2 is involved in these cancers include interacting with the cell cycle, ferroptosis, endoplasmic reticulum stress, reactive oxygen species, and so on [30, 46–50]. Here, our results proposed abnormal LDs accumulation as a tumor promoting effector and overexpression of LDs coat protein PLIN2 as a risk factor for OSCC prognosis. DGAT1 is the rate-limited enzyme that esterifies diglycerides to triglycerides [51]. Inhibition of DGAT1 decreased LDs density and affected the migration of prostate cancer cells [52]. Consistently, inhibition of DGAT1 in OSCC cells within our study also blocked the formation of LDs and attenuated their migration and invasion, which further verified the essential role of LDs biogenesis in cancer progression.

Evidence that EMT plays a role in tumor metastasis is solid and mounting. Through EMT, cancer cells reverse to an undifferentiated state and turn into the invasive phenotype characterized by loss of cell–cell contacts and enhanced motility [35, 37]. Increasing evidence suggests the connection between cancer lipid metabolism and the induction of EMT [28]. Accumulation of LDs promoted EMT and the subsequent lymph node metastasis in cervical cancer while paired related homeobox 1 (PRRX1) ‐induced EMT activated the reprogramming of FAs metabolism contributing to the enhanced invasion and metastasis in salivary adenoid cystic carcinoma [33, 53]. In the present study, we observed that PLIN2 depletion or overexpression regulates the expression of EMT markers, such as E-cadherin, N-cadherin, Vimentin, and Snail, which implied the influence of LDs density on the occurrence of the EMT. Phosphoinositide 3-kinase (PI3K)/AKT/mTOR is an important pathway positively regulating the process of EMT [54, 55]. Further exploration showed that overexpression of PLIN2 activated the PI3K pathway in the OSCC cells while pharmaceutical inhibitors of both PI3K and mTOR decreased the migration and invasion of OSCC cells significantly in vitro. These results elucidated the molecular mechanisms by which PLIN2-dependent LDs accumulation promoted the migration and invasion of OSCC cells.

Other than the verified influence of PLIN2 on the occurrence of EMT in the OSCC cells, our study further came up with the idea that overexpression of PLIN2 might be involved in the regulation of intracellular lipid peroxide accumulation and ferroptosis, given that two kinds of chemotherapy drugs possessing higher sensitivity in OSCC patients with high PLIN2 expression were both inhibitors of GPX4. Inhibition of GPX4 activity would lead to the accumulation of lipid peroxides, which in turn induced ferroptosis [56]. Although this mechanism needs further experimental verification, it also provides a possible theoretical reference for the adjuvant therapy targeting PLIN2 in OSCC patients, especially those with the burden of obesity.

## Conclusions

Collectively, these results indicated the promoting effect of FAs on the migration and invasion of OSCC cells. PLIN2-dependent LDs accumulation mediated this effect via the activation of the PI3K/AKT/mTOR pathway, ultimately leading to enhanced EMT of OSCC cells. Besides, overexpression of PLIN2 increased the metastasis of OSCC cells in the animal model and predicted poor prognosis in OSCC patients. Therefore, to ameliorate metastasis, the dietary intervention of OSCC patients should take FAs into consideration, and PLIN2-targeting treatment might provide new therapeutic insights for OSCC patients, especially those obese. More studies are necessary to deeply understand the lipid metabolism of OSCC both big picture and detail.

## Materials and methods

### Cell culture and reagents

The human OSCC cell line CAL33 was originally purchased from the Leibniz Institute DSMZ-German Collection of Microorganisms and Cell Cultures GmbH (Braunschweig, Germany) while the cell lines SCC9 and SCC25 were purchased from ATCC (Rockville, MD, USA). The cell lines HSC3, HSC4, and HSC6 were provided by J. Silvio Gutkind (NIH, Bethesda, MD, USA). The cell lines used in this study were authenticated using short tandem repeat (STR) analysis and regularly tested for mycoplasma. HSC3, HSC4, HSC6, and CAL33 cells were cultured in Dulbecco’s Modified Eagle Medium (DMEM, Gibco, USA) with 10% fetal bovine serum (FBS, Front Biomed, USA) and 1% penicillin/streptomycin (PS, Gibco, USA). SCC9 and SCC25 cells were maintained in DMEM/F12 (1:1) (Gibco, USA) supplemented with 10% FBS, 1% PS and 0.4 μg/ml hydrocortisone. All cells were cultured at 37 °C in a 5% CO_2_ atmosphere. Experiments were performed with cells undergoing logarithmic growth. PA and OA were provided by Sigma–Aldrich. BEZ235 (Selleck Chemicals, USA) was dissolved in dimethyl sulfoxide (MP Biomedicals, USA) at 1 mM as a stock solution.

### RNA sequencing and data analysis

HSC3 cells treated with 25 μM PA for 24 h were washed with Phosphate buffered saline (PBS, Solarbio, Beijing) and then lysed using Trizol (Invitrogen, USA) for 15 min under room temperature. The lysed samples were stored in an RNase-free eppendorf tube under −80 °C before transferring to Guangdong Longsee Biomedical Co., Ltd for RNA sequencing. The sequencing data were then processed and further analyzed. Firstly, a threshold of adjusted *P* < 0.05 and |log_2_FC | > 1 was set to indicate significant differentially expressed genes (DEGs). Subsequently, the biological process Gene Ontology (GO) analysis and the KEGG pathway analysis were conducted utilizing the online website of KOBAS (http://kobas.cbi.pku.edu.cn/genelist/) according to the standard protocols.

### Stable transduction with lentivirus

Human PLIN2-overexpression lentiviral vectors were constructed, identified, and supplied by Shanghai Genechem Chemical Technology Co., Ltd (Genechem, Shanghai, China). To be specific, the cDNA of PLIN2 was sub-cloned using Taq DNA polymerase (SinoBio Biltech Co. Ltd, Shanghai, China) and inserted into the BamHI/AgeI sites of GV260 lentivirus vectors (Genechem, Shanghai, China). After identification of the correct sequence and lentivirus packaging, HSC3 and CAL33 cells were infected at a multiplicity of infection (MOI) equal to 10 for 12 h. Then these cells were further treated with 2 mg/ml puromycin for 2 weeks to establish stable cell lines and maintained with 1 mg/ml puromycin thereafter. The transfection efficiency of PLIN2 overexpression was confirmed by RT-PCR and western blot analysis.

### Transient transfection with small interfering RNA

To knock down the expression of PLIN2, small interfering RNA (siRNA) sequences specifically targeting PLIN2 (si-PLIN2) and negative control siRNA (si-NC) were chemically synthesized by RiboBio (Guangzhou, China). The sequences targeting PLIN2 were as follows: siRNA#1 (GACTGCCTATTCTGAATCA), siRNA#2 (GTCACGTACTCTTGCAATT). For transfection, HSC6 cells were seeded in 6-well plates at 50-70% confluence. Cells were transfected with 100 pM siRNA sequences using RNAiMAX (Invitrogen, USA) following the manufacturer’s specifications. Cells were used for subsequent assays at the indicated time after transfection. The knockdown of diacylglycerol O-acyltransferase 1 (DGAT1) in HSC3 cells adopted the same protocols as above. The sequences targeting DGAT1 were as follows: siRNA#1 (CTGTGGTCTTACTGGTTGA), siRNA#2 (CCCGGTTATTTCTGGAGAA).

### Wound-healing assay

Human OSCC cell lines were seeded in 6-well plates (5 × 10^5^ cells/well) and treated with the indicated concentration of PA/OA for 24 h after the cells adhered to the bottom. Fatty acid free bovine serum albumin -V (BSA-V)(Solarbio, Beijing) was used as solvent control. When a monolayer of cells had spread over the bottom of the well, a 20 μl pipette tip was used to vertically scratch the cell layer in the middle of the bottom. After washing the cell suspension and cell fragments with PBS, the cells were cultured in the serum-free medium to allow the wound to heal. Phase contrast images of the same position were taken under an inverted microscope (Carl Zeiss AG, Germany) at 0 and 24 h. The wound healing ratio of 3 independent experiments was calculated using ImageJ.

### Transwell migration and invasion assay

Cell migration and invasion assays were performed using 24-well plates and 8 μm transwell inserts (Falcon, USA). For migration assays, OSCC cells were treated with the indicated concentration of PA/OA or fatty acid free BSA-V for 24 h after the cells adhered to the bottom. Subsequently, 4 × 10^4^ –3 × 10^5^ pretreated cells were re-suspended in 200 μl of serum-free media and seeded in the upper chamber. Then, 800 μl of medium containing 10% FBS was added to the lower chamber. For invasion assays, every single insert was coated with 50 μl Matrigel matrix (Corning, USA) diluted with serum-free DMEM (1:9) and kept in a humidified incubator at 37 °C with 5% CO_2_ for at least 2 h before adding in 6 × 10^4^ –3 × 10^5^ pretreated cells. After being cultured for 24 h, cells remaining in the upper inserts were removed with cotton swabs. The migrated/invasive cells on the lower side of the inserts were fixed in 4% paraformaldehyde and stained with 0.5% crystal violet for 30 min at room temperature. Five visual fields were randomly selected under an inverted microscope (Carl Zeiss AG, Germany) to calculate the cell number of 3 independent experiments.

### Cell Counting Kit-8 (CCK-8) proliferation assay

Cell proliferation was analyzed using the CCK-8 (Dojindo, Japan) assay. In brief, 1 × 10^3^ cells were seeded in 96-well plates, allowed to adhere overnight, and then treated with media containing the indicated concentration of PA/OA or fatty acid free BSA-V. Cell viability was determined at the baseline and every 24 h. Specifically, 10 μl CCK-8 mixed with 90 μl serum-free DMEM was added to each well, and plates were incubated at 37 °C for 1 h. Then, the absorbance was measured at 450 nm using a microplate reader (BioTek Instruments, USA) with light avoided. All experiments were conducted in triplicate.

### RNA extraction and Quantitative real-time PCR (qRT-PCR)

Total RNA of cells was extracted by RNA quick extraction kit (YiShan Biotech, Shanghai) and reverse transcribed by HiScript III RT SuperMix (Vazyme, Nanjing). qRT–PCR (Universal SYBR, Vazyme) was performed on an ABI QuantStudio5 system (ABI, USA) to quantify mRNA levels. All results were normalized to GAPDH. The primers being used were demonstrated in Table 1.

**Table 1:** The primers used in qRT-PCR.

*GAPDH* forward	5’- CTCCTCCTGTTCGACAGTCAGC -3’
*GAPDH* reverse	5’- CCCAATACGACCAAATCCGTT -3’
*PLIN2* forward	5’- TTGCAGTTGCCAATACCTATGC -3’
*PLIN2* reverse	5’- CCAGTCACAGTAGTCGTCACA -3’
*DGAT1* forward	5’- TATTGCGGCCAATGTCTTTGC -3’
*DGAT1* reverse	5’- CACTGGAGTGATAGACTCAACCA -3’
*CDH1* forward	5’- CTCCACAGCCACCGTCACC -3’
*CDH1* reverse	5’- GCCCACGCCAAAGTCCTCG-3’
*CDH2* forward	5’- CTTCATTATCAACCCCATCTCG -3’
*CDH2* reverse	5’- CTCAAATGAAACCGGGCTA -3’
*VIM* forward	5’- GATGTTTCCAAGCCTGACCT -3’
*VIM* reverse	5’- GTACCATTCTTCTGCCTCCTG-3’
*SNAI1* forward	5’- CAGACCCACTCAGATGTCAAG -3’
*SNAI1* reverse	5’- CTTGTGGAGCAGGGACATT-3’

### Protein extraction and Western blot (WB) analysis

Cells were collected and lysed in radio immunoprecipitation assay (RIPA) buffer (ComWin Biotech, Beijing) supplemented with 1% protease and phosphatase inhibitors (ComWin Biotech, Beijing). The bicinchoninic acid (BCA) protein assay kit (ComWin Biotech, Beijing) was used to measure the concentrations of the lysates. The lysates were then incubated at 100 °C for 10 min and mixed with a loading buffer (4:1; ComWin Biotech, Beijing). Equal amounts (30 µg/lane) of protein were loaded onto a 10% sodium dodecyl sulfate polyacrylamide gel electrophoresis (SDS-PAGE) gel for separation and then transferred to a 0.22 μm polyvinylidene fluoride (PVDF) membrane (Merck Millipore, Germany). The membrane was blocked in 5% skim milk powder (BioFroxx, Germany) for 1 h at room temperature and then incubated with primary antibodies overnight at 4 °C. Subsequently, the membrane was washed in tris-buffered saline with tween-20 (TBST) 3 times and incubated with HRP-conjugated goat anti-rabbit/mouse secondary antibodies (Emarbio Science & Technology Company, Beijing) for 1 h at room temperature. The signal was visualized using an enhanced chemiluminescence substrate (Merck Millipore, Germany). Similar results were obtained from 3 independent experiments. The primary antibodies included PLIN2 (1:500) purchased from Novus Biologicals; mammalian target of rapamycin (mTOR) (1:1000), E-Cadherin (1:1000), Snail (1:1000) purchased from Cell Signaling Technology; and PI3K (1:1000), phosphorylated mTOR (p-mTOR) (1:2000), protein kinase B (AKT) (1:1000), phosphorylated AKT (p-AKT) (1:2000), N-Cadherin (1:2000), Vimentin (1:10000), glyceraldehyde-3-phosphate dehydrogenase (GAPDH) (1:20000) purchased from Proteintech Group.

### Lipid droplets staining assay

Intracellular LDs were measured using the cell permeable fluorescent lipophilic dye, BODIPY 493/503 (Thermo Fisher Scientific, USA). Cells (1 × 10^5^) were seeded in the glass plate designed for confocal (35 mm, Biosharp Life Science, Anhui). After overnight incubation and pretreatment with indicated compounds, cells were incubated with 4 μl BODIPY diluted in 1 ml PBS for 25 min at room temperature. Following nuclei counterstain with 1 μl Hoechst 33342 (Invitrogen, USA) diluted in 1 ml PBS for 15 min at room temperature and washed with PBS, digital images were obtained by the laser scanning confocal microscope (Carl Zeiss AG, Germany).

### Tumor metastasis assay in nude mice

BALB/c nude mice (6–8 weeks, 18–22 g, male) were purchased from the Experimental Animal Center at Sun Yat-sen University Eastern Campus and housed in the Experimental Animal Center at Sun Yat-sen University North Campus. HSC3 cells (1.5 × 10^6^) stably overexpressing PLIN2 were resuspended in 200 μl PBS and subsequently injected into the tail veins of nude mice (15 mice/group). The in vivo metastasis and colonization of tumor cells were observed by the in vivo fluorescence imaging system (PerkinElmer, USA) immediately, 2 weeks, and 4 weeks after the injection. To be specific, 1 g luciferase substrate D (-) -Luciferin powder was fully dissolved in 66.667 ml Dulbecco’s PBS (D-PBS) buffer in advance and stored at −20 °C in the dark. A total of 200 μl thawed luciferase substrate solution was intraperitoneally injected into the nude mice 15 min before the detection. After inhalation anesthesia with isoflurane, they were placed in the imaging system to acquire fluorescence images.

The mice were euthanized 7 weeks after injection for tissue retrieval. The lungs were removed and embedded in paraffin to histopathologically examine the metastatic locus via hematoxylin and eosin (H&E) staining. Slides were examined with an APERIO AT2 (Leica, Germany) and images were captured using Aperio ImageScope software. Histopathological diagnosis was performed by an experienced pathologist.

For immunohistochemical analysis, paraffin embedding sections were deparaffinized and rehydrated in a series of gradient alcohols. Then, slides were immersed in sodium citrate buffer and boiled in a microwave for antigen retrieval. After inhibition of endogenous peroxidases and blocking, samples were covered with a primary antibody against pan-cytokeratin (PAN-CK) (ZSGB-BIO, Beijing) or other primary antibodies used for the western blot analysis mentioned above. After incubation at 4 °C overnight, a universal HRP-conjugated goat anti-rabbit/mouse secondary antibody in diaminobenzidine (DAB) Detection Kit (Gene Tech, Shanghai) was applied for 30 min and then visualized with DAB chromogenic agent. Nuclear counterstaining was performed using hematoxylin.

### Bioinformatics analysis of PLIN2 expression

Gene expression data and corresponding patient clinical data for OSCC were downloaded from The Cancer Genome Atlas (TCGA) database (https://portal.gdc.cancer.gov/) and the Gene Expression Omnibus (GEO) database. A total of 328 OSCC cases were obtained from TCGA and 253 from GSE65858 (https://www.ncbi.nlm.nih.gov/geo/geo2r/?acc=GSE65858). The R package “survminer” was adopted to determine the optimal cutoff dividing the patients into high expression or low expression groups. Survival curves were plotted by the Kaplan–Meier method and compared by the log-rank test. We also combined the information in the TCGA database and the Genomics of Drug Sensitivity in Cancer (GDSC) database to select chemotherapy drugs more sensitive to patients with high PLIN2 expression [57]. To further explore the biological pathways involved in PLIN2 regulation, we performed the correlation analysis between PLIN2 and other genes in TCGA OSCC dataset. A gene set enrichment analysis (GSEA) was conducted with genes possessing a high correlation coefficient by the ClusterProfiler R package with adjusted *P* < 0.05 to analyze their enrichment of the ‘Hallmark’ gene set.

### Statistical analysis

Statistical analysis was performed using GraphPad Prism 5.0 (GraphPad Software, USA). Quantitative data were expressed as the mean ± standard error (SE) of the independently repeated experiments indicated in each method described above. The data were first tested for the normality and homogeneity of variance. Student’s t-test or One-way ANOVA was used to compare the differences between two or more groups when appropriate. The chi-square test was adopted to compare the metastasis rate between the two groups. *P* < 0.05 was considered statistically significant.

## Supplementary information

Supplementary figure 1

Supplementary figure 2

Supplementary figure 3

Supplementary figure 4

Supplementary figure 5

Supplementary figure legends

Supplementary Table 1

original western blot figure

## Data Availability

The datasets used and/or analyzed during the current study are available from the corresponding author on reasonable request.
