# Supplementary figures and images for: Perilipin2-dependent lipid droplets accumulation promotes metastasis of oral squamous cell carcinoma via epithelial-mesenchymal transition
Source: Cell Death Discov. 2025 Jan 28;11:30. doi: 10.1038/s41420-025-02314-1 (PMC11775315; doi:10.1038/s41420-025-02314-1)

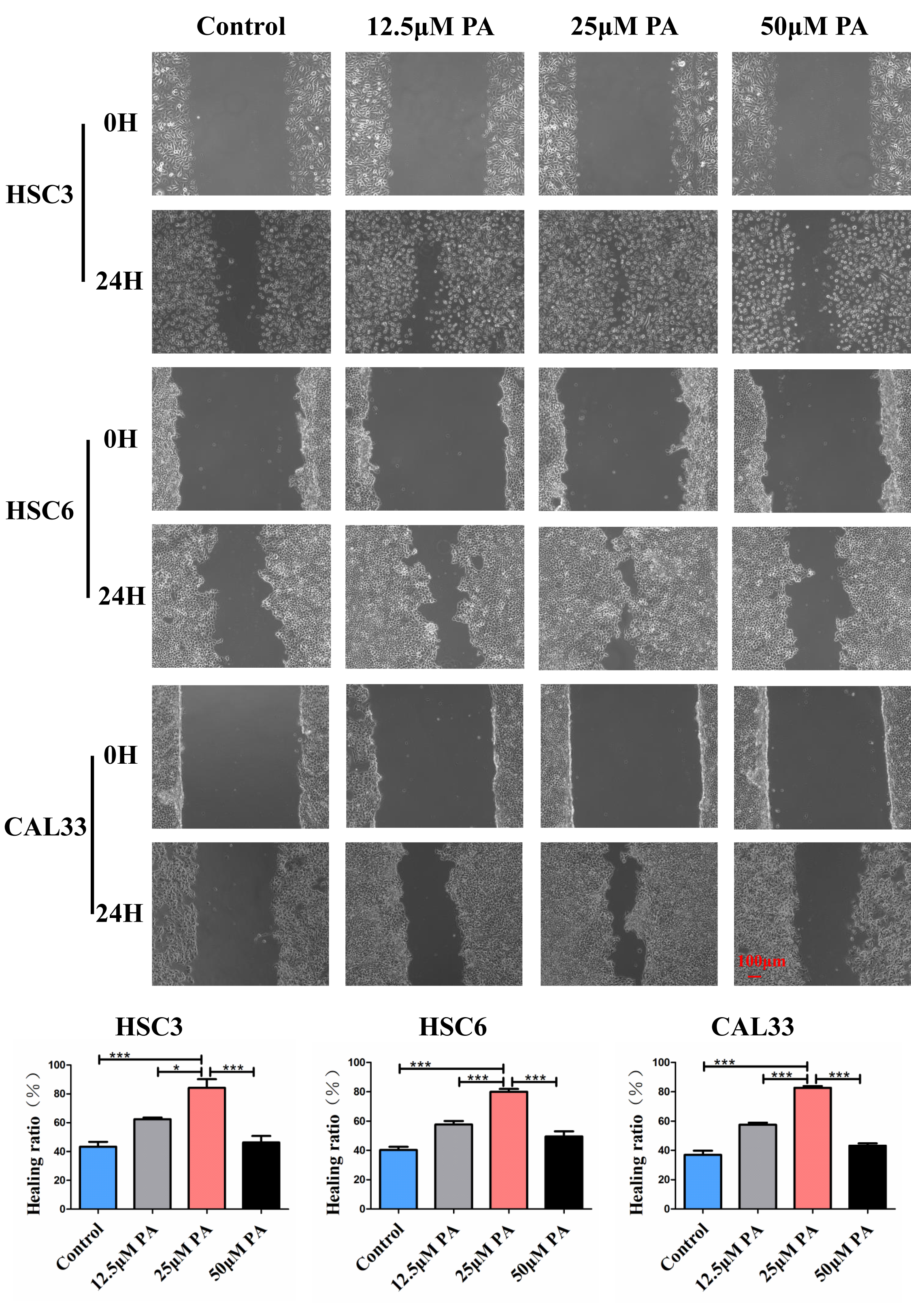

Supplement: Supplementary file 1 — Supplementary figure 1 [file 41420_2025_2314_MOESM1_ESM.tif]

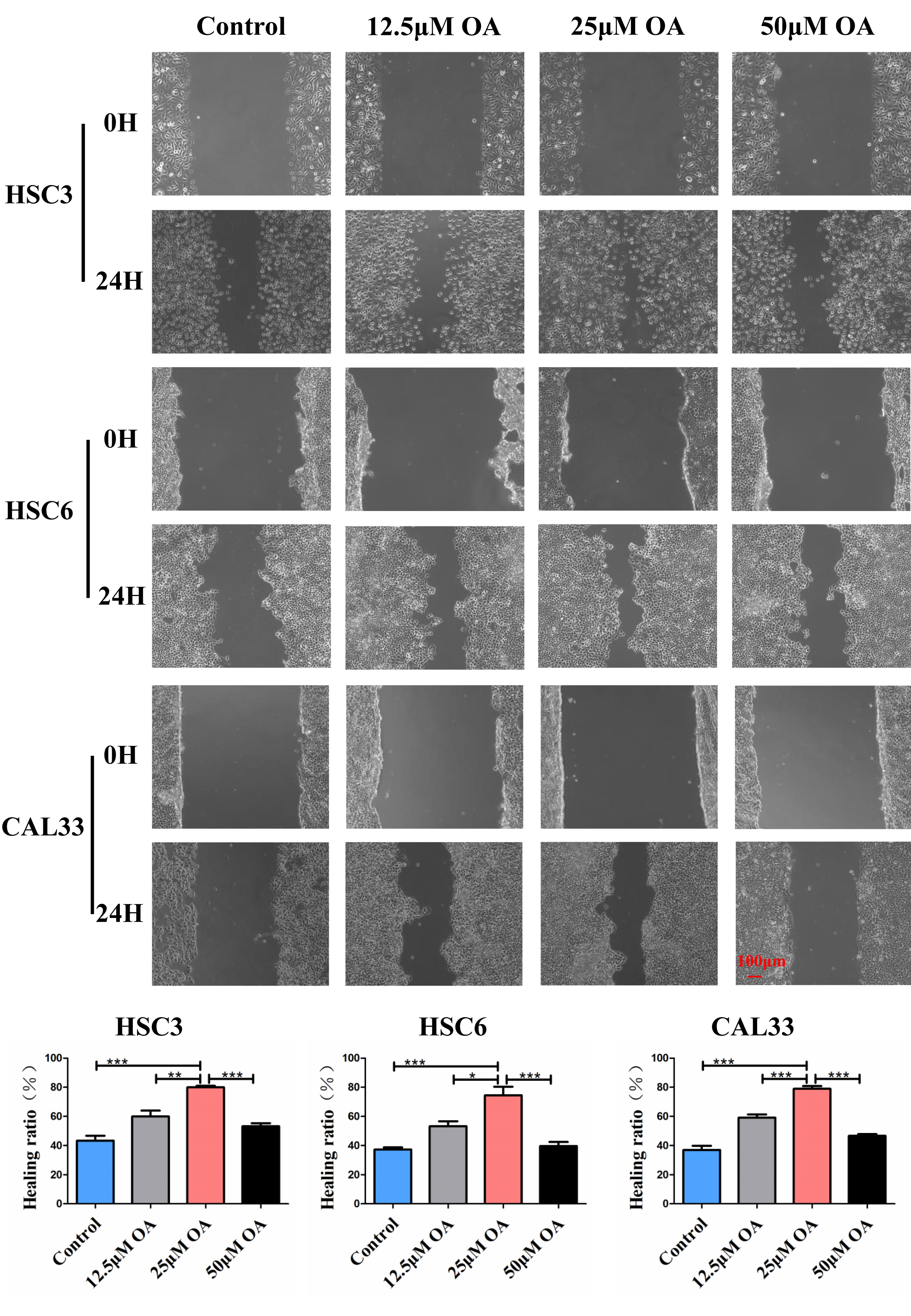

Supplement: Supplementary file 2 — Supplementary figure 2 [file 41420_2025_2314_MOESM2_ESM.tif]

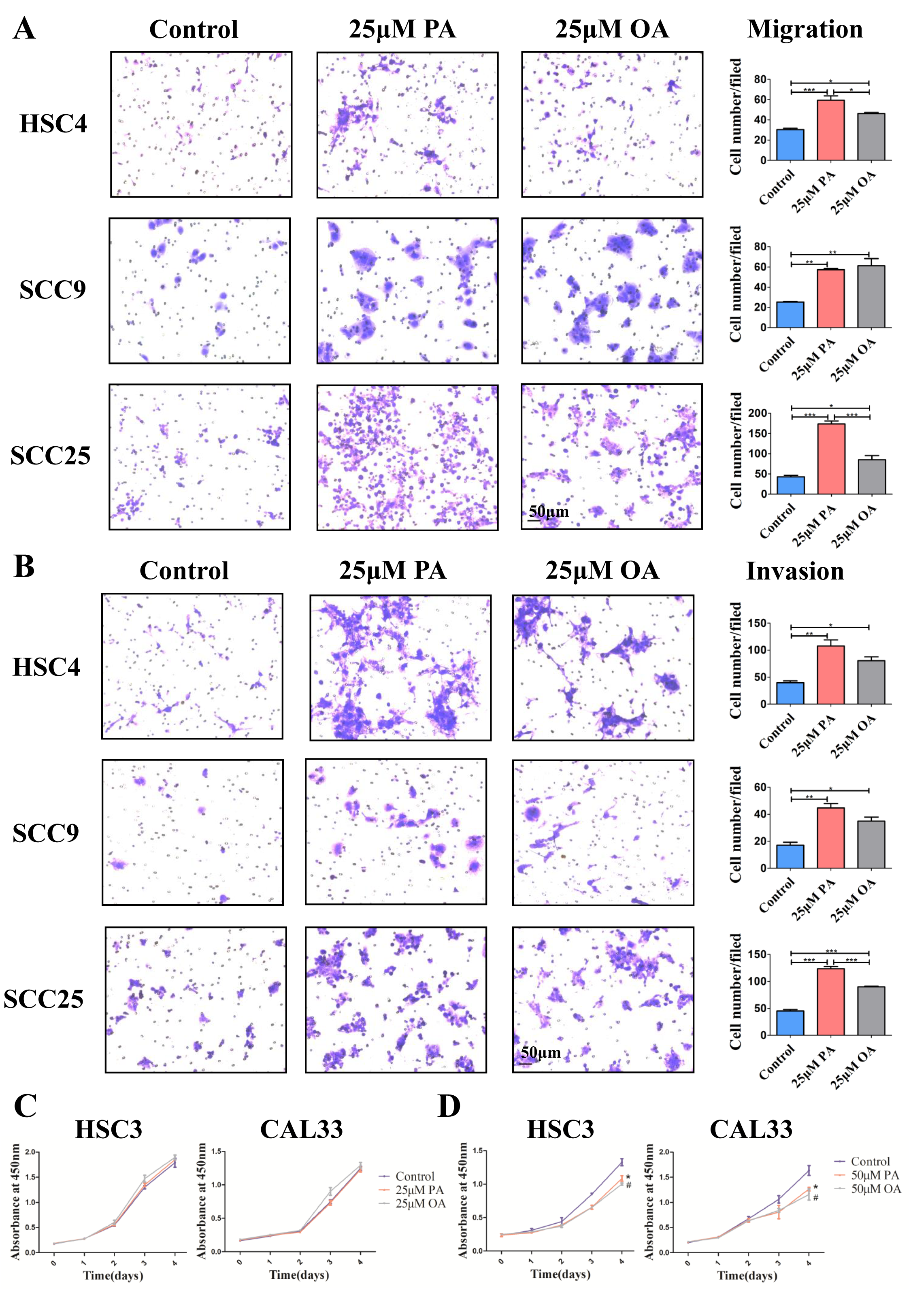

Supplement: Supplementary file 3 — Supplementary figure 3 [file 41420_2025_2314_MOESM3_ESM.tif]

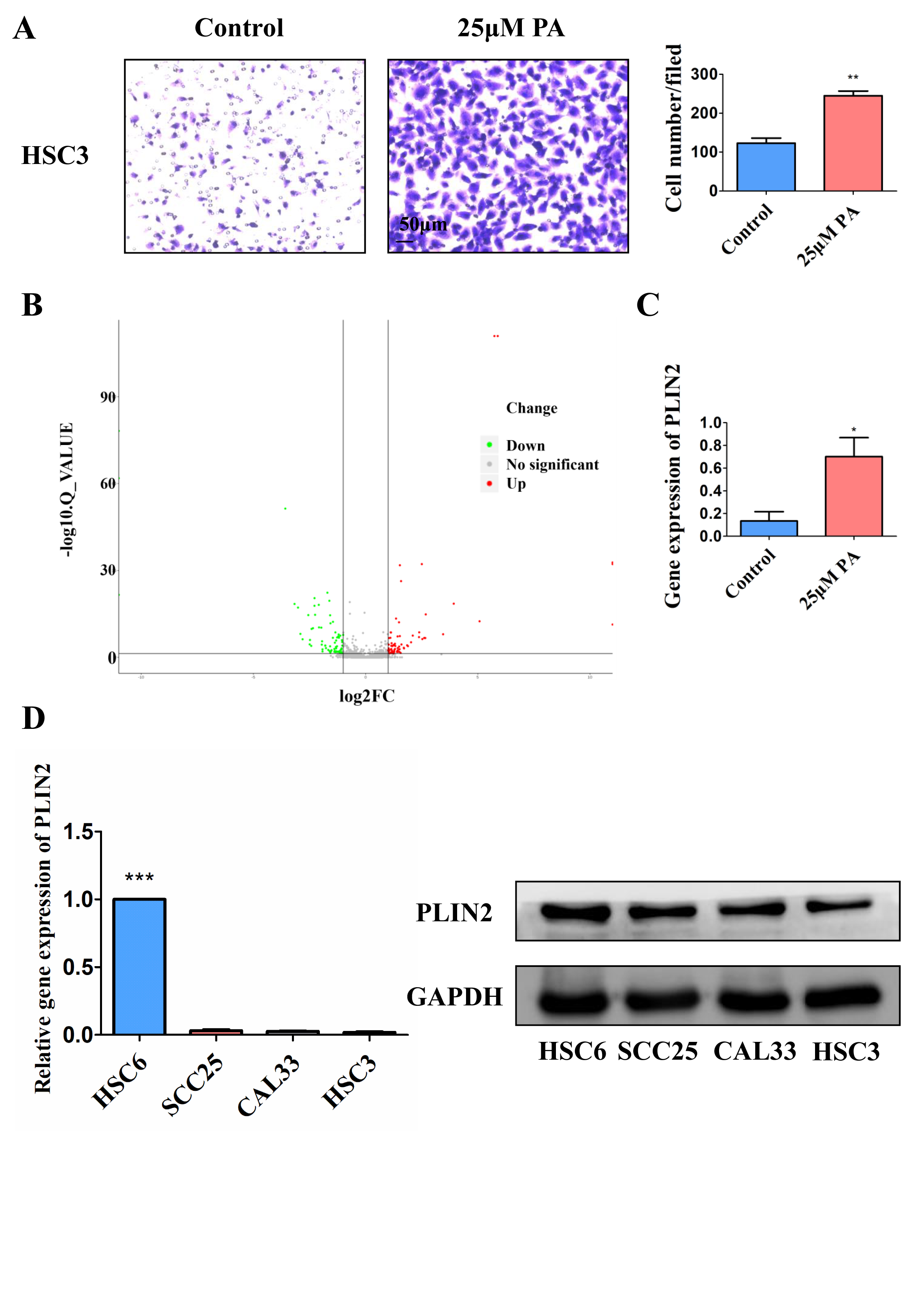

Supplement: Supplementary file 4 — Supplementary figure 4 [file 41420_2025_2314_MOESM4_ESM.tif]

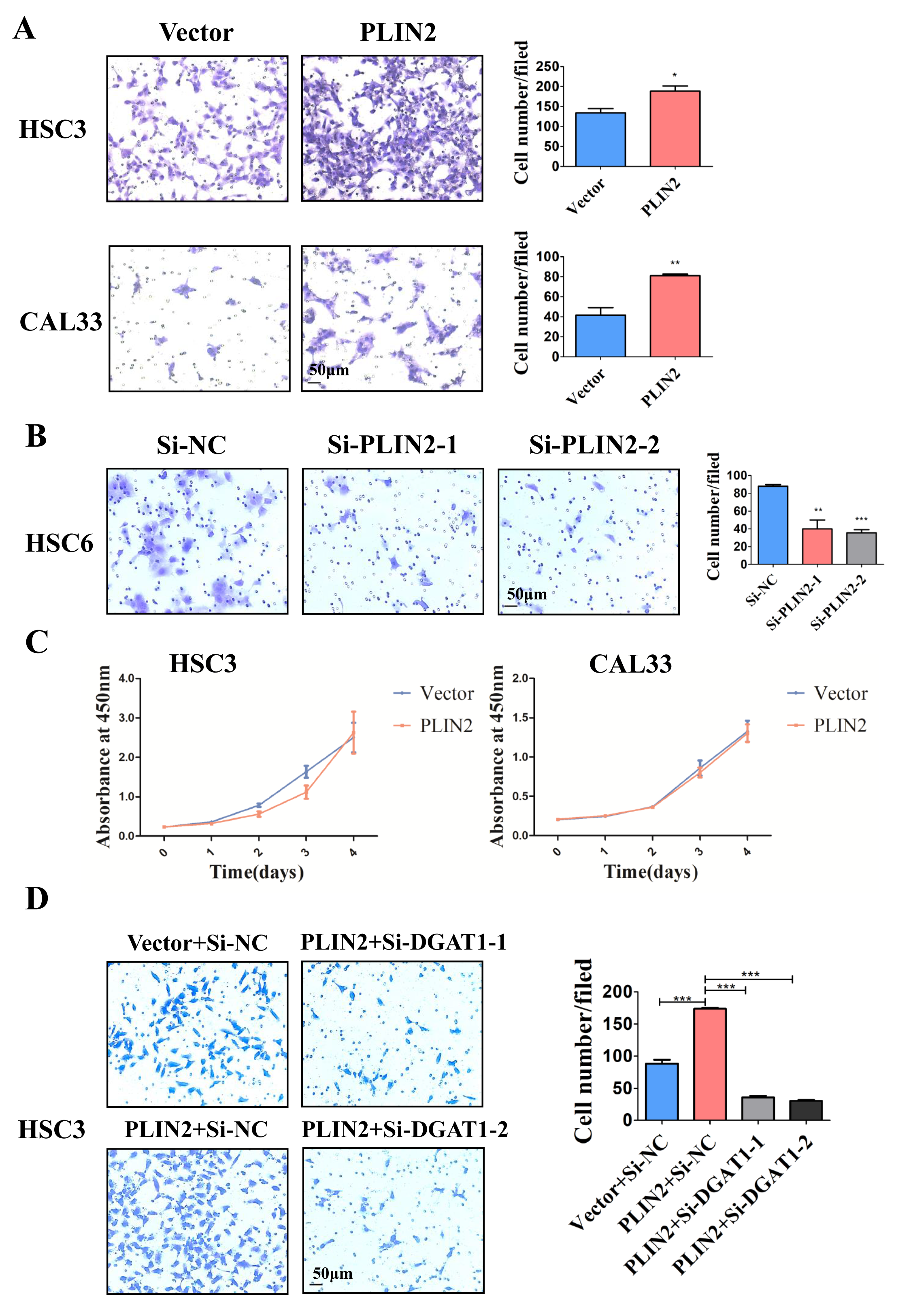

Supplement: Supplementary file 5 — Supplementary figure 5 [file 41420_2025_2314_MOESM5_ESM.tif]
