# Supplementary material for: Perilipin2-dependent lipid droplets accumulation promotes metastasis of oral squamous cell carcinoma via epithelial-mesenchymal transition
Source: Cell Death Discov. 2025 Jan 28;11:30. doi: 10.1038/s41420-025-02314-1 (PMC11775315; doi:10.1038/s41420-025-02314-1)
Supplement: Supplementary file 6 — Supplementary figure legends [file 41420_2025_2314_MOESM6_ESM.docx]

**Supplementary figure 1 Effects of different concentrations of PA stimulation on the wound healing ability of different OSCC cells.** **P* < 0.01, ****P* < 0.001. PA: Palmitic acid. Bar: 100 μm.

**Supplementary figure 2 Effects of different concentrations of OA stimulation on the wound healing ability of different OSCC cells.** **P* < 0.05, ***P* < 0.01, ****P* < 0.001. OA: Oleic acid. Bar: 100 μm.

**Supplementary figure 3 FAs stimulation can promote the migration and invasion of OSCC cells but has no significant effect on proliferation.** (A-B) Transwell results showed that 25 μM PA or OA stimulation increased the *in vitro* migration and invasion ability of different OSCC cell. (C) CCK-8 results showed that 25 μM PA or OA stimulation had no significant effect on cell viability of OSCC cells *in vitro.* (D) CCK-8 results showed that 50 μM PA or OA stimulation significantly reduced the cell viability of OSCC cells *in vitro*. Bar: 50 μm. **P* < 0.05, ***P* < 0.01, ****P* < 0.001, # no siginificance. FAs, Fatty acids; PA: Palmitic acid; OA: Oleic acid.

**Supplementary figure 4 PLIN2 is a potential effector molecule of PA promoting the migration of OSCC cells *in vitro*.** (A) Transwell results confirmed that PA stimulation in the same batch of RNA sequencing samples effectively increased the migration ability of HSC3 cells *in vitro*; (B) The volcanic map of the differentially expressed genes within the sequencing results; (C) The expression of PLIN2 in OSCC cells after PA stimulation was significantly upregulated within the sequencing results; (D) qRT-PCR and WB results indicated that the background expression of PLIN2 varied in different OSCC cells. Bar: 50 μm ***P* < 0.01, ****P* < 0.001. PA: Palmitic acid; NC: Negative control.

**Supplementary figure 5 PLIN2 can promote the migration of OSCC cells but has no significant effect on proliferation.** (A) Transwell results confirmed that overexpression of PLIN2 can increase the *in vitro* migration ability of HSC3 and CAL33 cells. (B) Transwell results confirmed that knockdown of PLIN2 inhibited the *in vitro* migration ability of HSC6 cells. (C) CCK-8 results showed that overexpression of PLIN2 had no significant effect on the *in vitro* proliferation ability of HSC3 and CAL33 cells. (D) Transwell results confirmed that knockdown of DGAT1 significantly decreased the *in vitro* migration ability of HSC3 cells stably overexpressing PLIN2. Bar: 50 μm. **P* < 0.05, ***P* < 0.01, ****P* < 0.001. NC: Negative control; LDs: Lipid droplets.
