# Supplementary material for: Perilipin2-dependent lipid droplets accumulation promotes metastasis of oral squamous cell carcinoma via epithelial-mesenchymal transition
Source: Cell Death Discov. 2025 Jan 28;11:30. doi: 10.1038/s41420-025-02314-1 (PMC11775315; doi:10.1038/s41420-025-02314-1)
Supplement: Supplementary file 8 — original western blot figure [file 41420_2025_2314_MOESM8_ESM.pptx]

## Slide 1
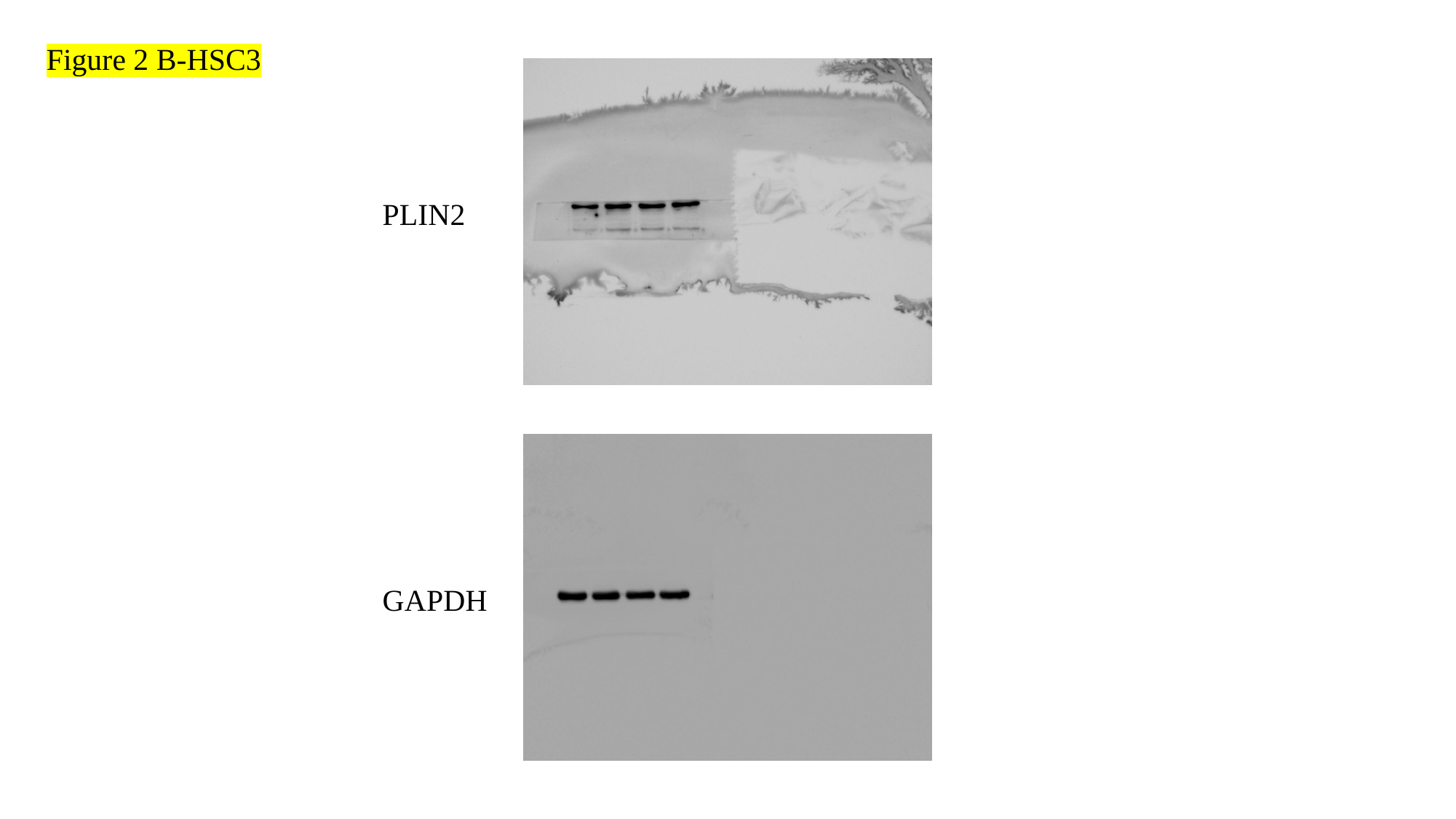

Figure 2 B-HSC3
PLIN2
GAPDH

## Slide 2
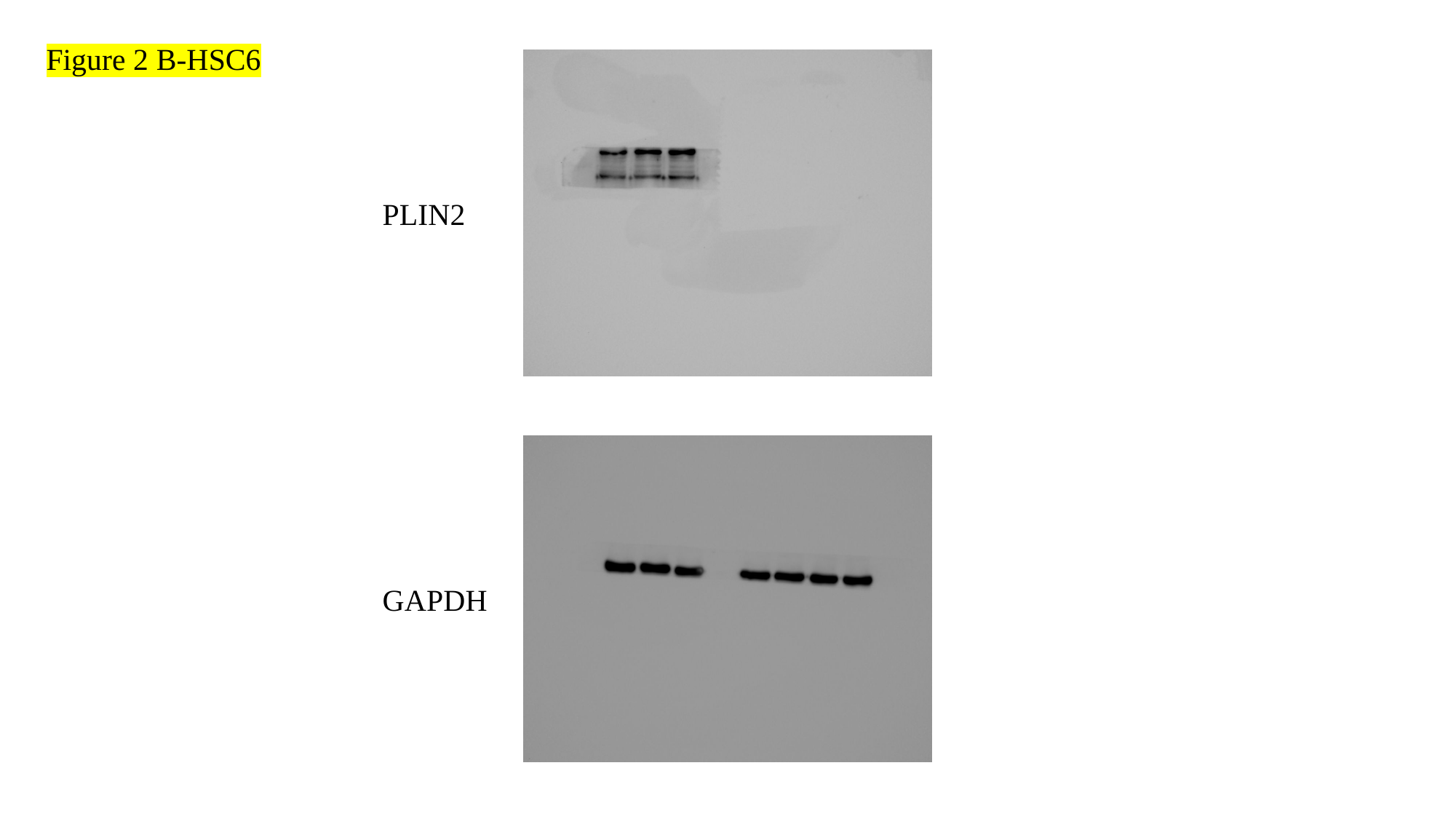

Figure 2 B-HSC6
PLIN2
GAPDH

## Slide 3
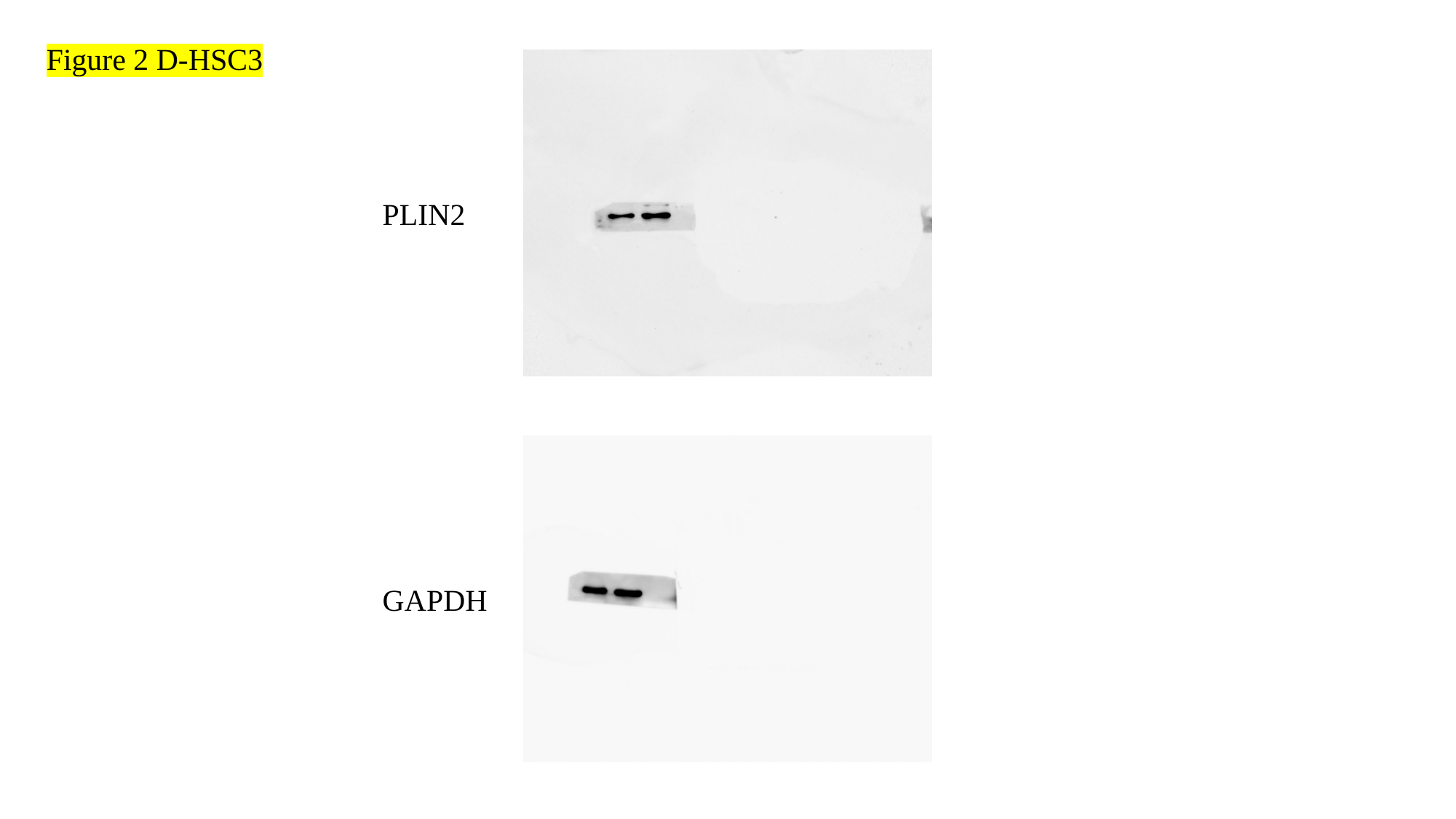

Figure 2 D-HSC3
PLIN2
GAPDH

## Slide 4
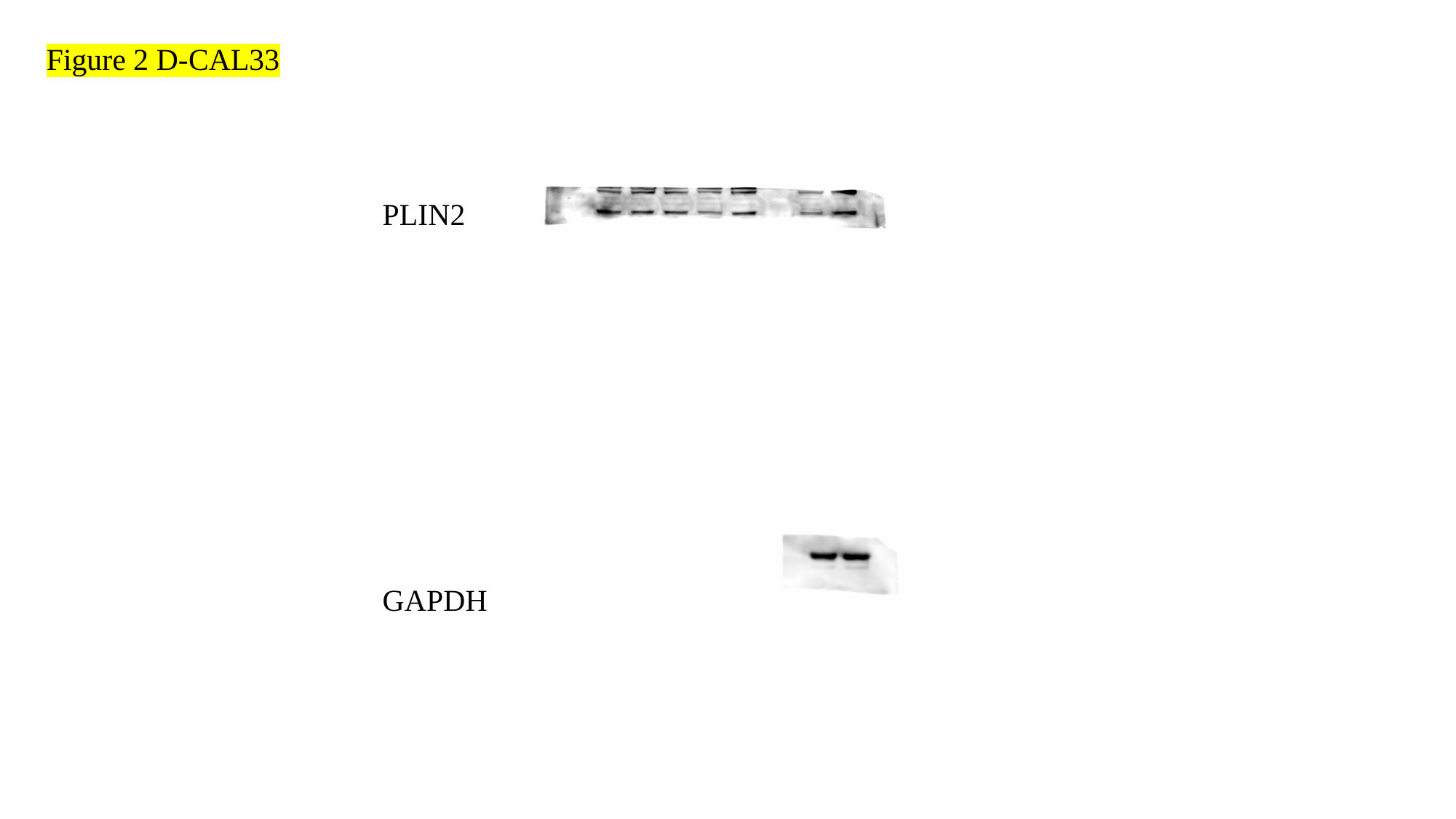

Figure 2 D-CAL33
PLIN2
GAPDH

## Slide 5
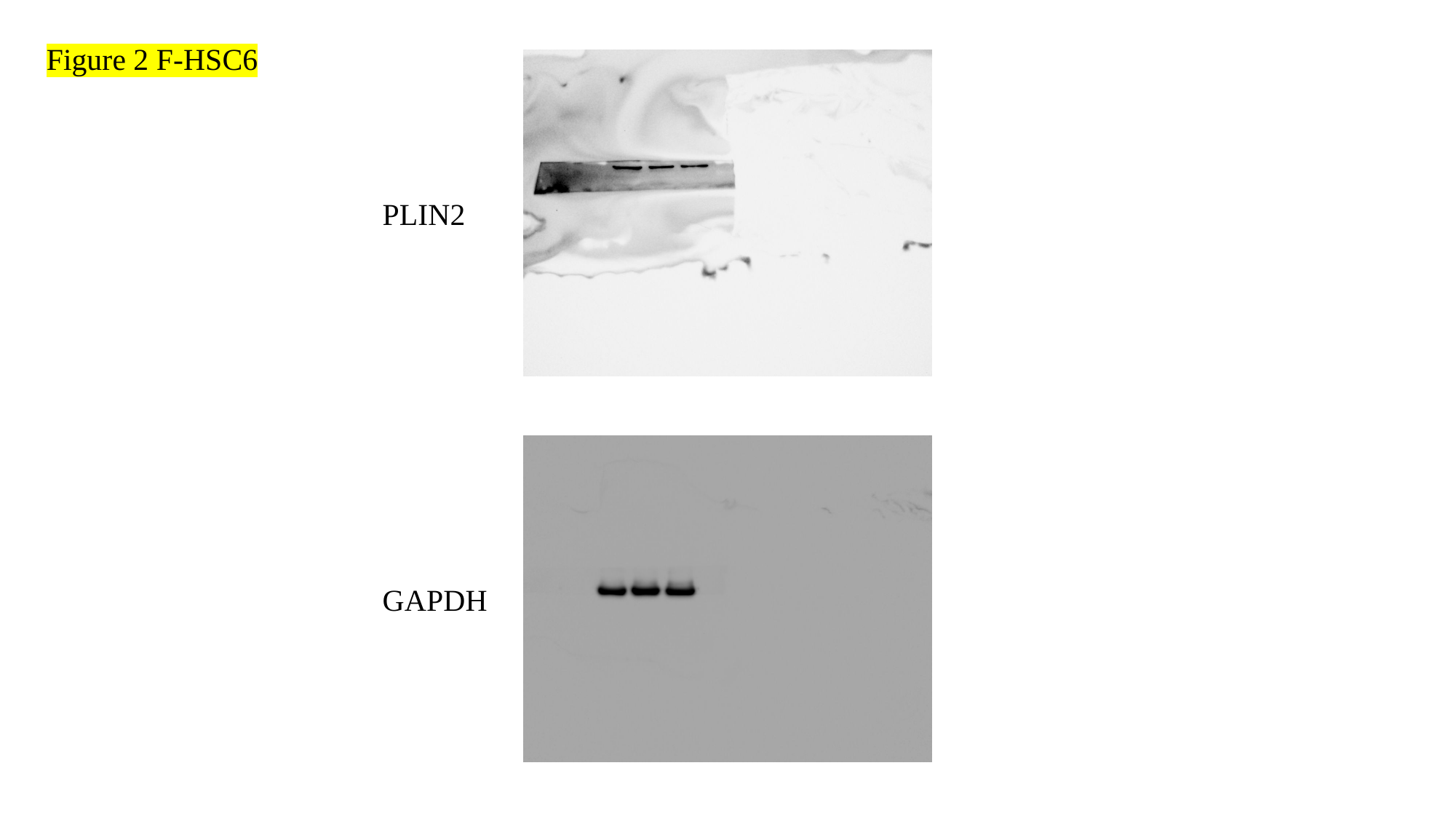

Figure 2 F-HSC6
PLIN2
GAPDH

## Slide 6
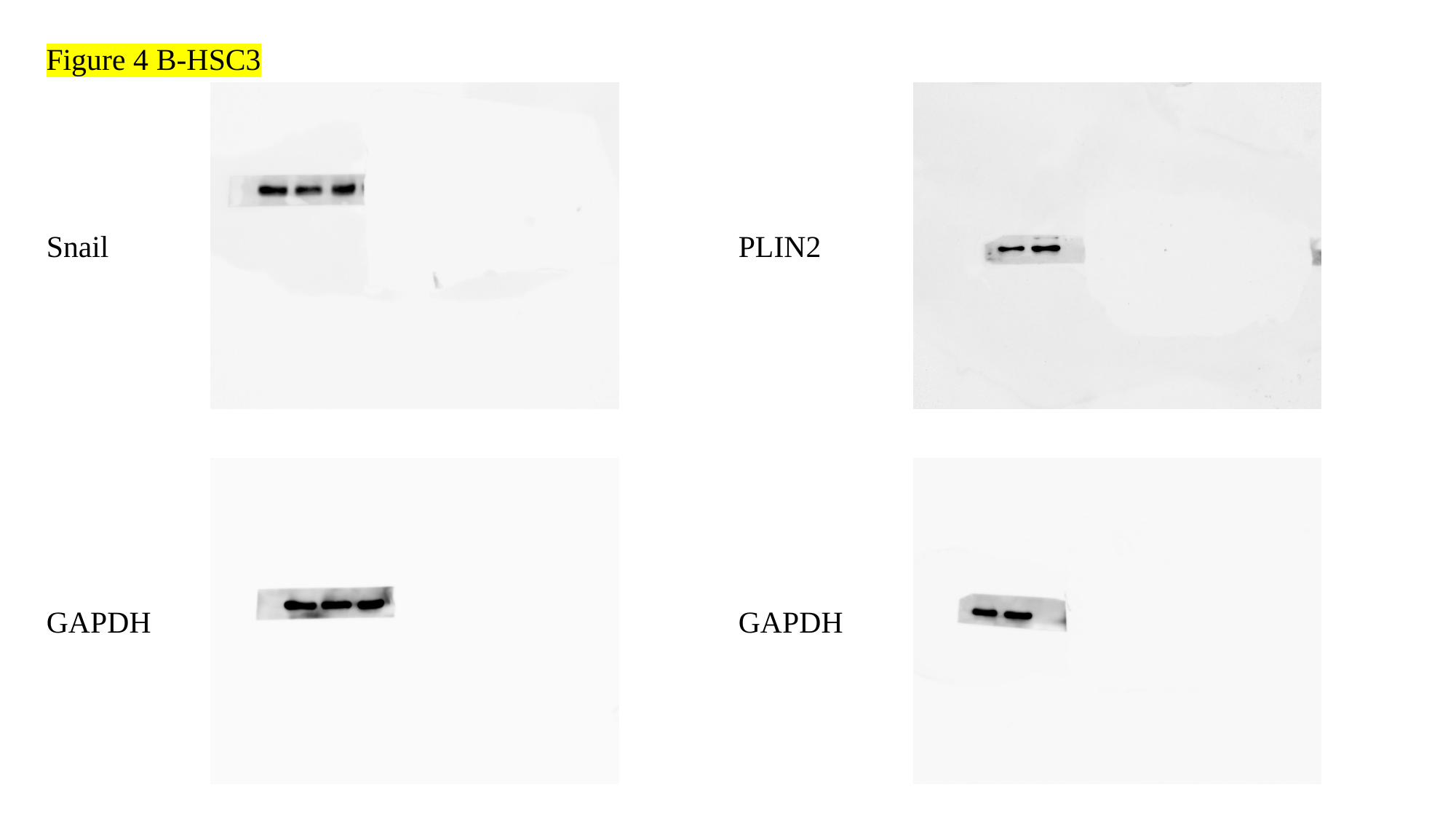

Figure 4 B-HSC3
Snail
PLIN2
GAPDH
GAPDH

## Slide 7
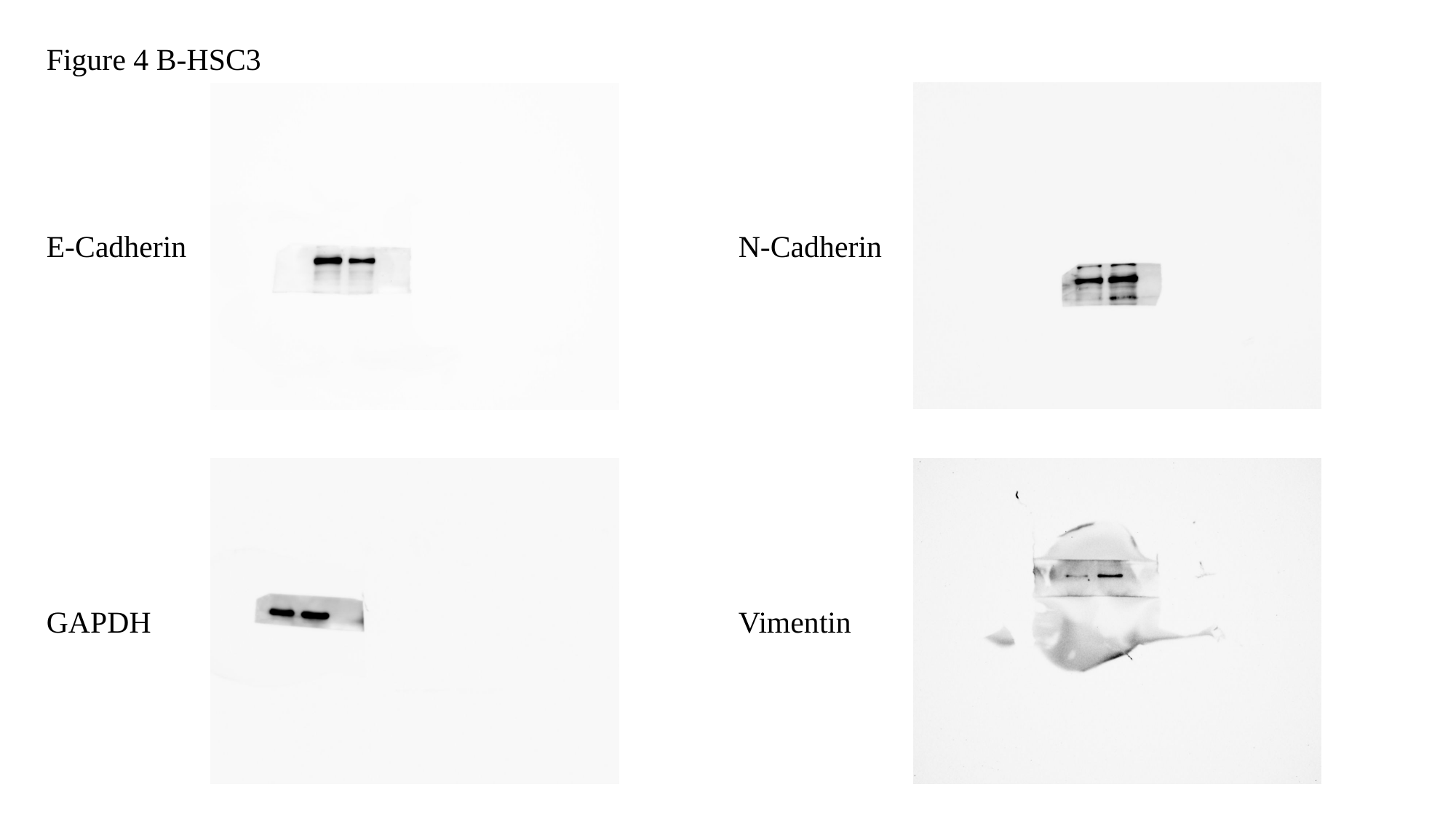

Figure 4 B-HSC3
E-Cadherin
N-Cadherin
GAPDH
Vimentin

## Slide 8
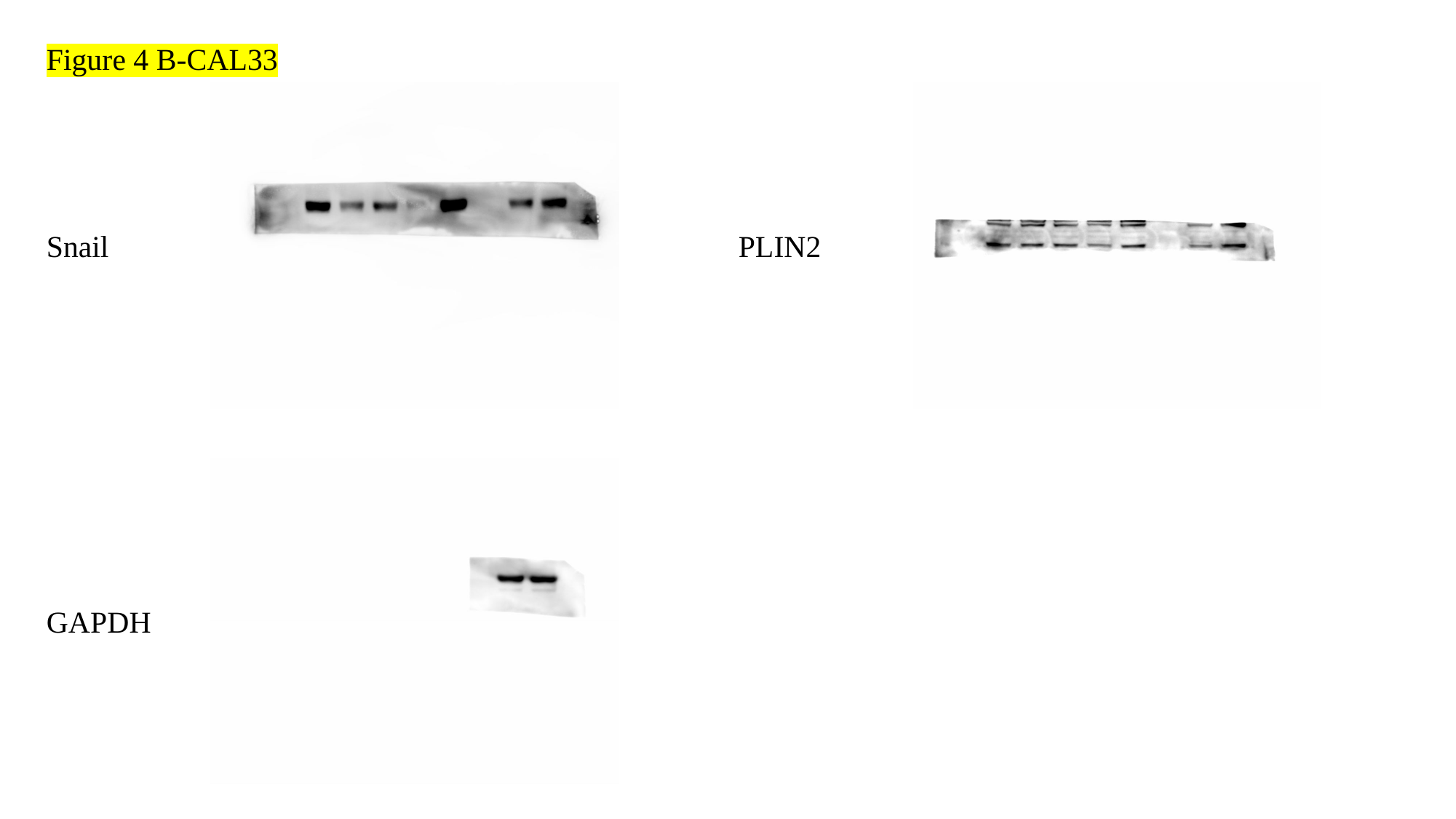

Figure 4 B-CAL33
Snail
PLIN2
GAPDH

## Slide 9
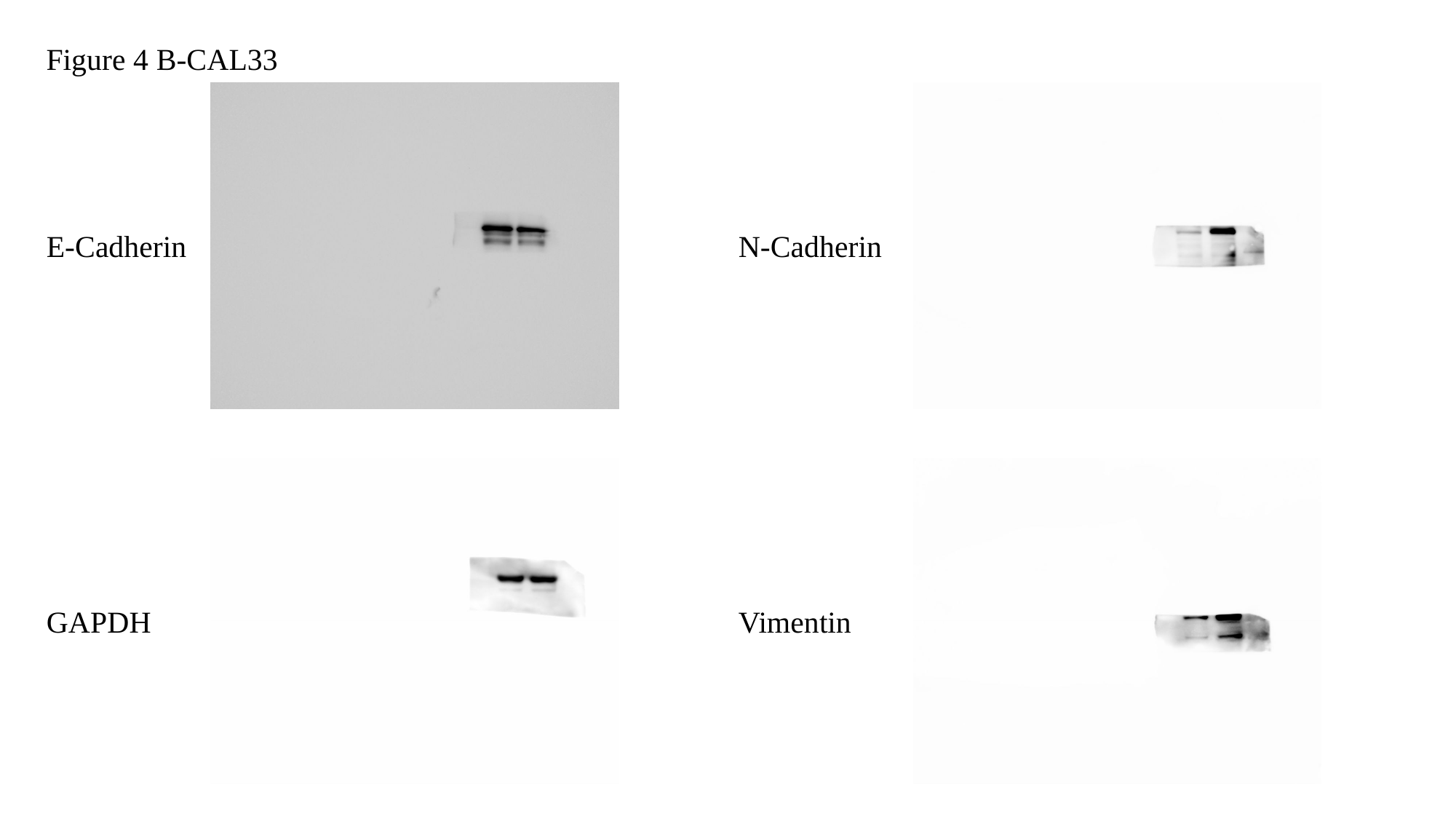

Figure 4 B-CAL33
E-Cadherin
N-Cadherin
GAPDH
Vimentin

## Slide 10
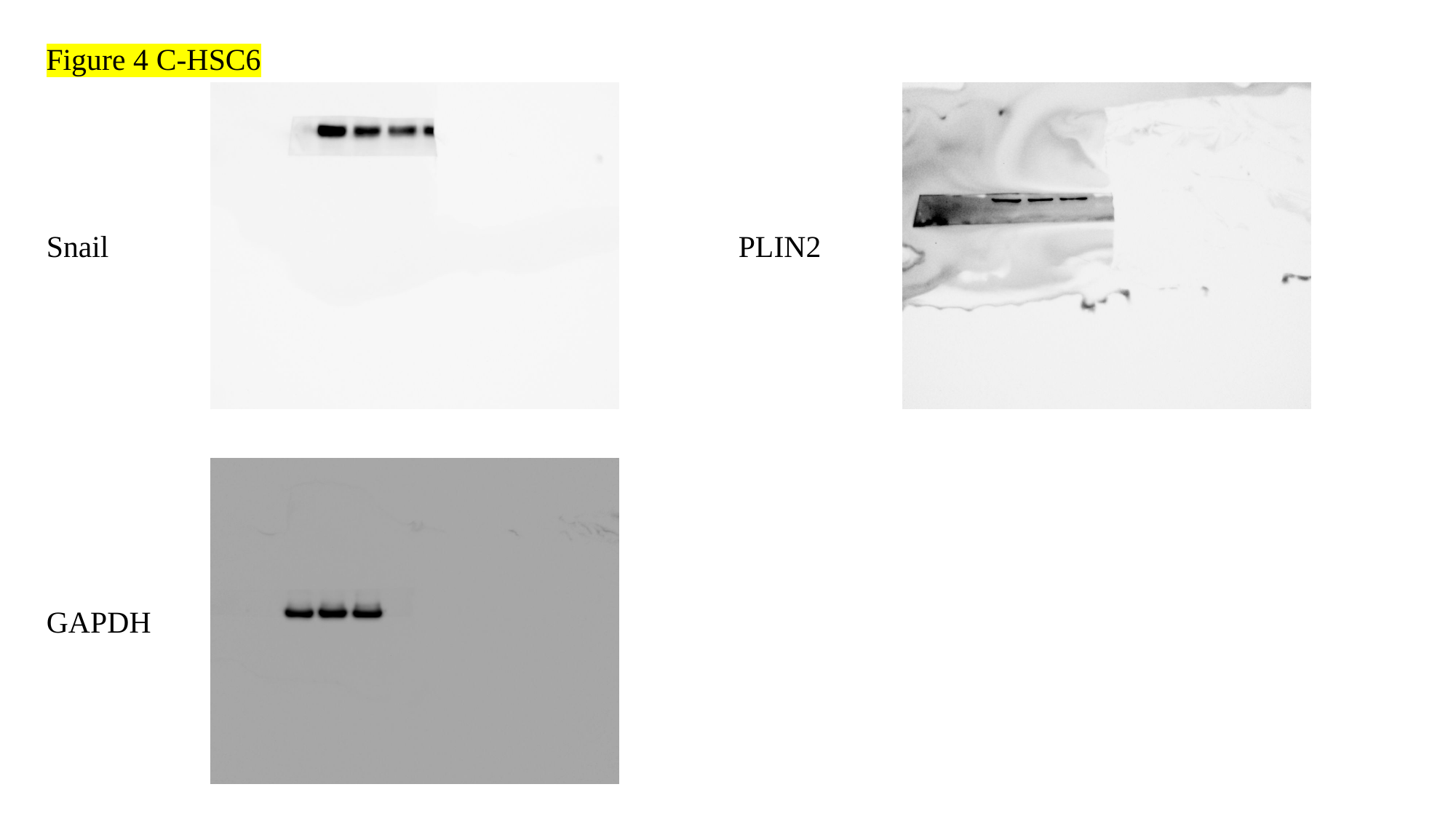

Figure 4 C-HSC6
Snail
PLIN2
GAPDH

## Slide 11
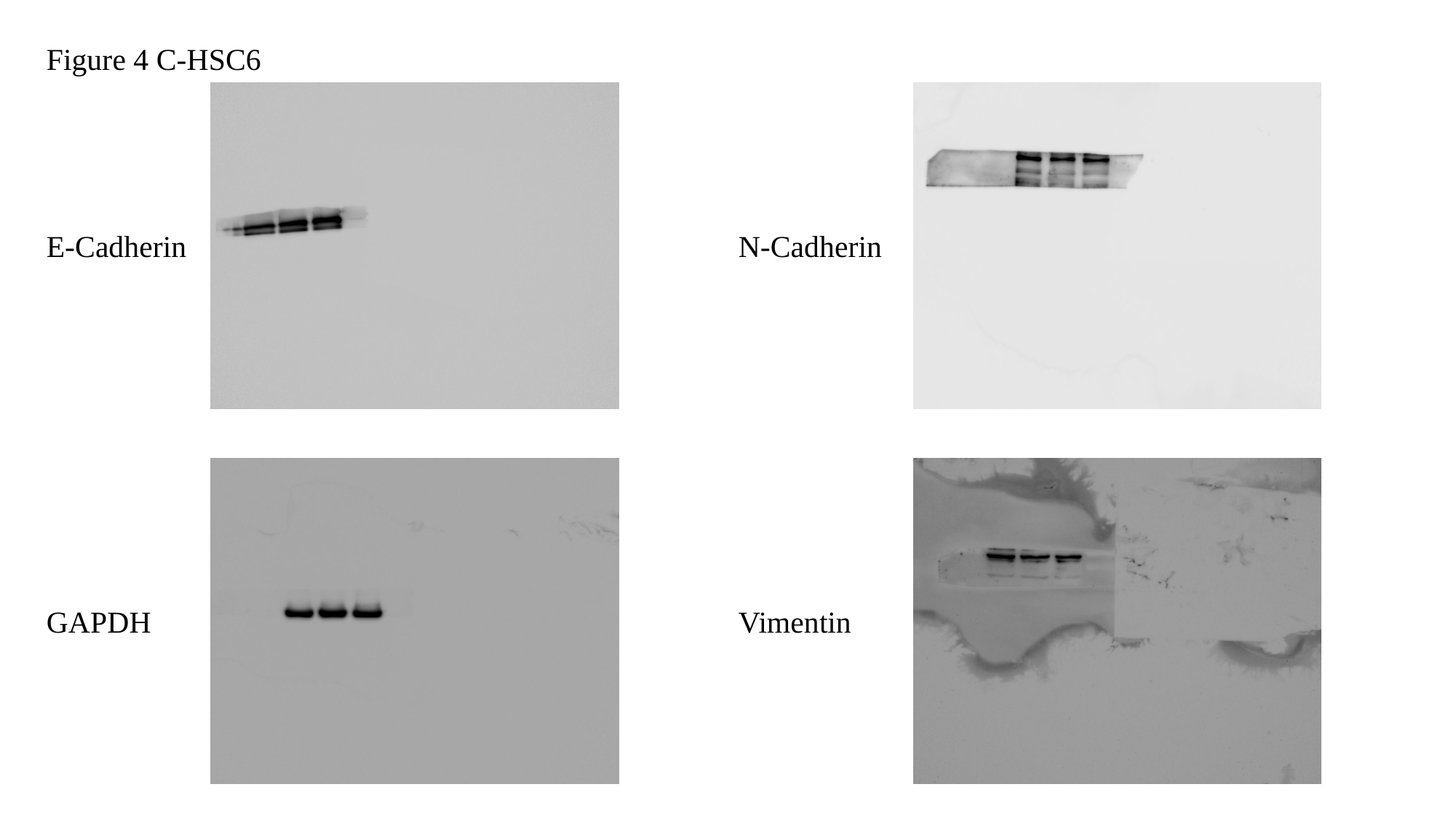

Figure 4 C-HSC6
E-Cadherin
N-Cadherin
GAPDH
Vimentin

## Slide 12
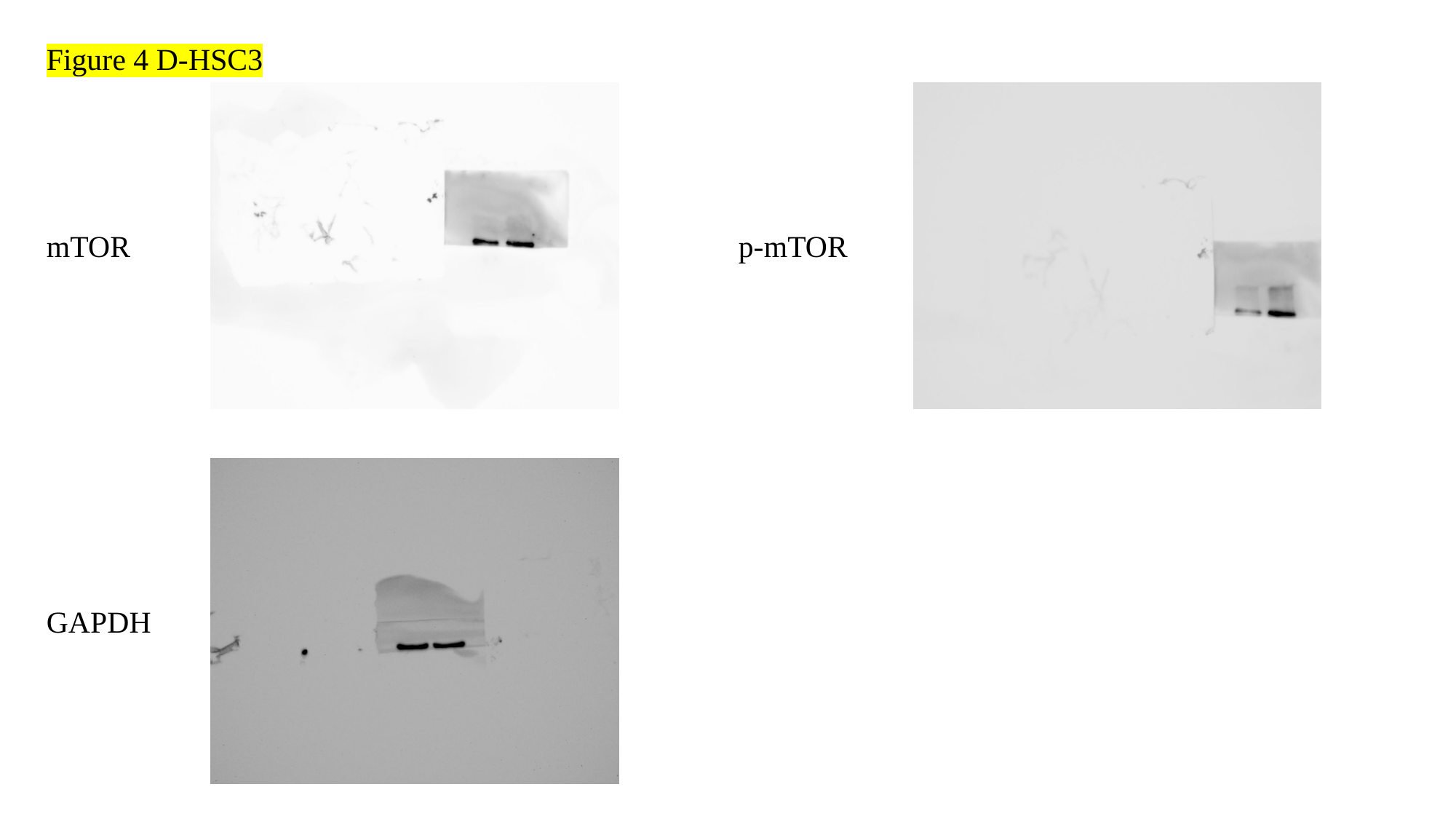

Figure 4 D-HSC3
mTOR
p-mTOR
GAPDH

## Slide 13
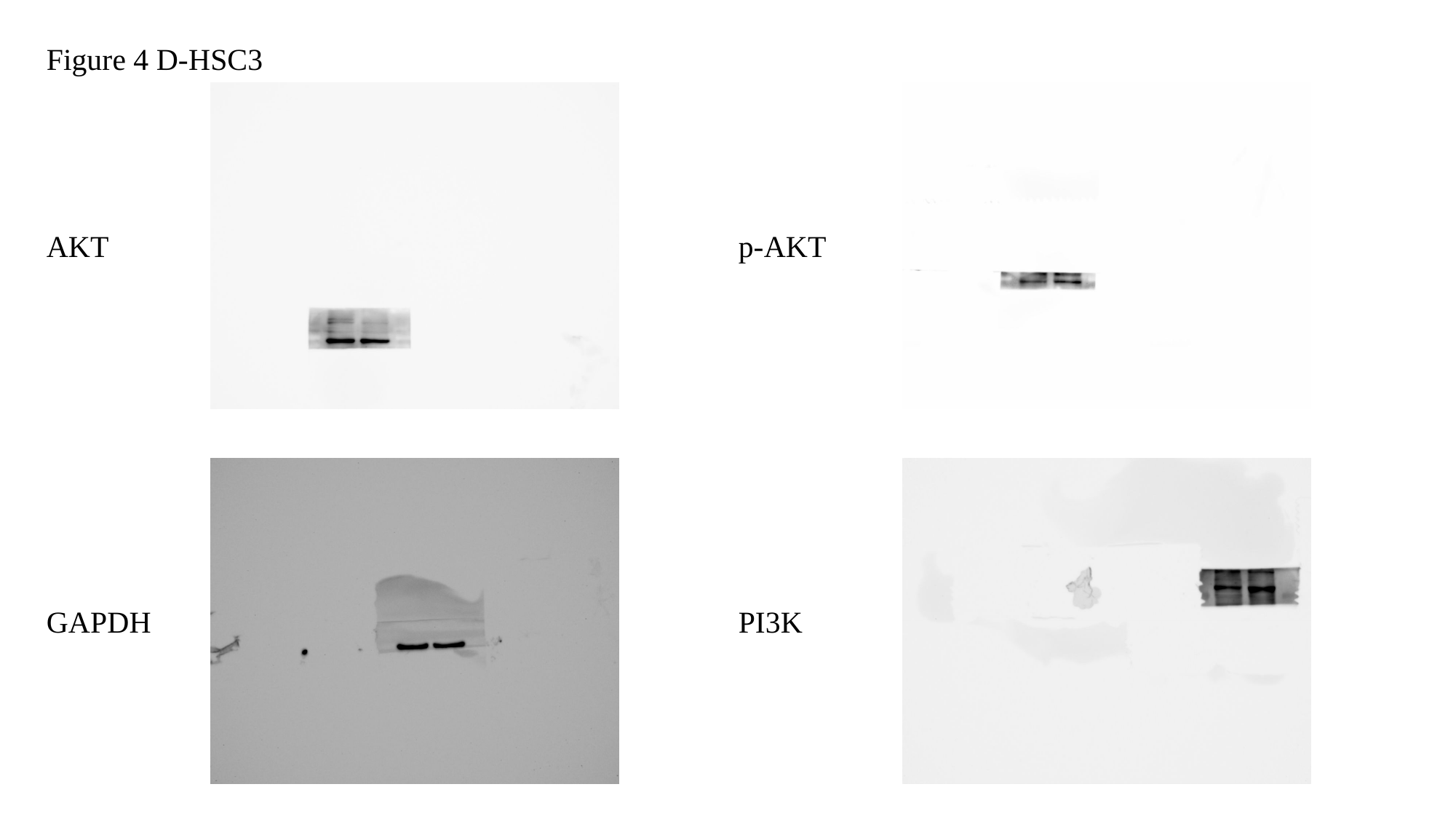

Figure 4 D-HSC3
AKT
p-AKT
GAPDH
PI3K

## Slide 14
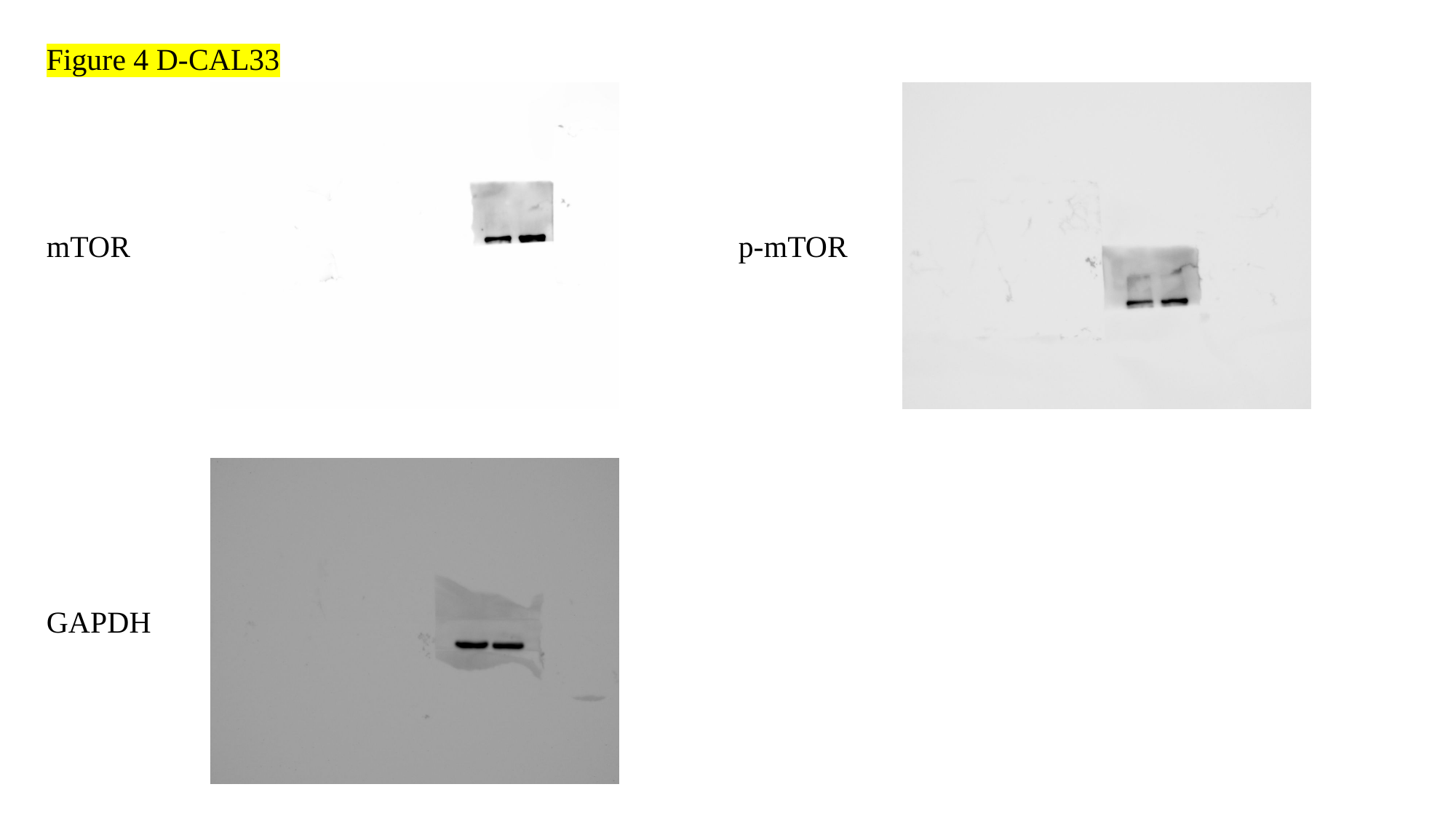

Figure 4 D-CAL33
mTOR
p-mTOR
GAPDH

## Slide 15
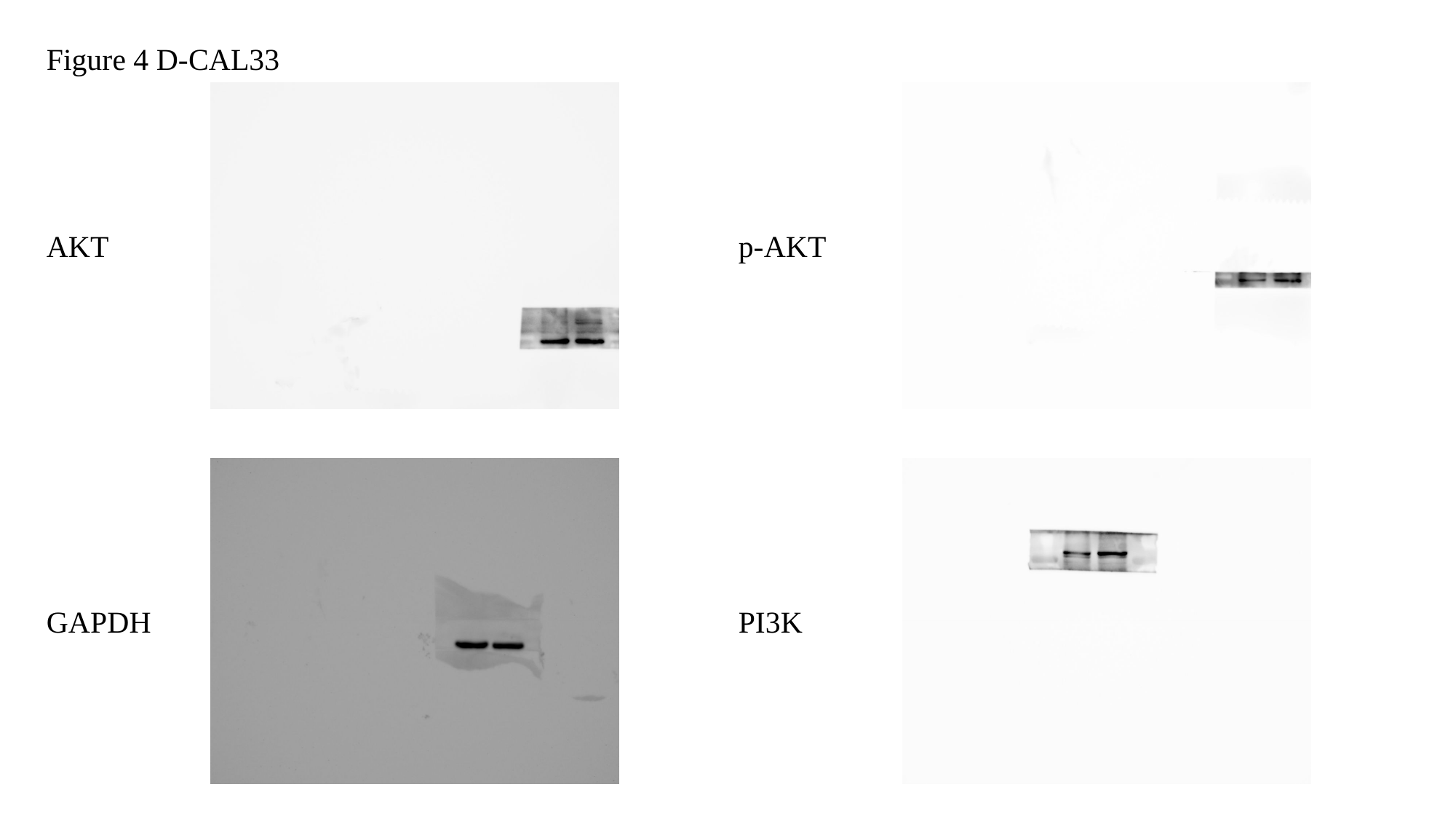

Figure 4 D-CAL33
AKT
p-AKT
GAPDH
PI3K

## Slide 16
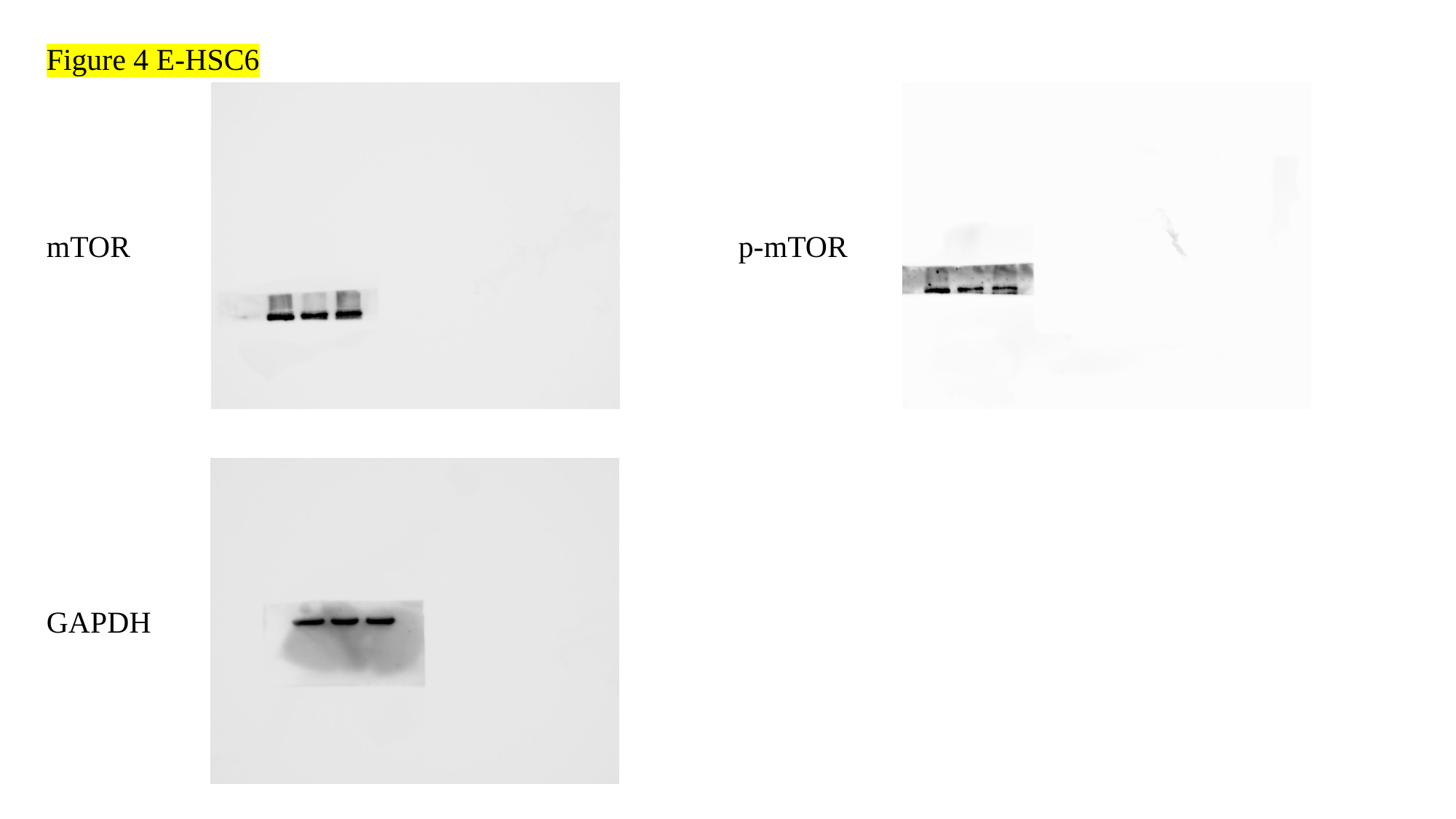

Figure 4 E-HSC6
mTOR
p-mTOR
GAPDH

## Slide 17
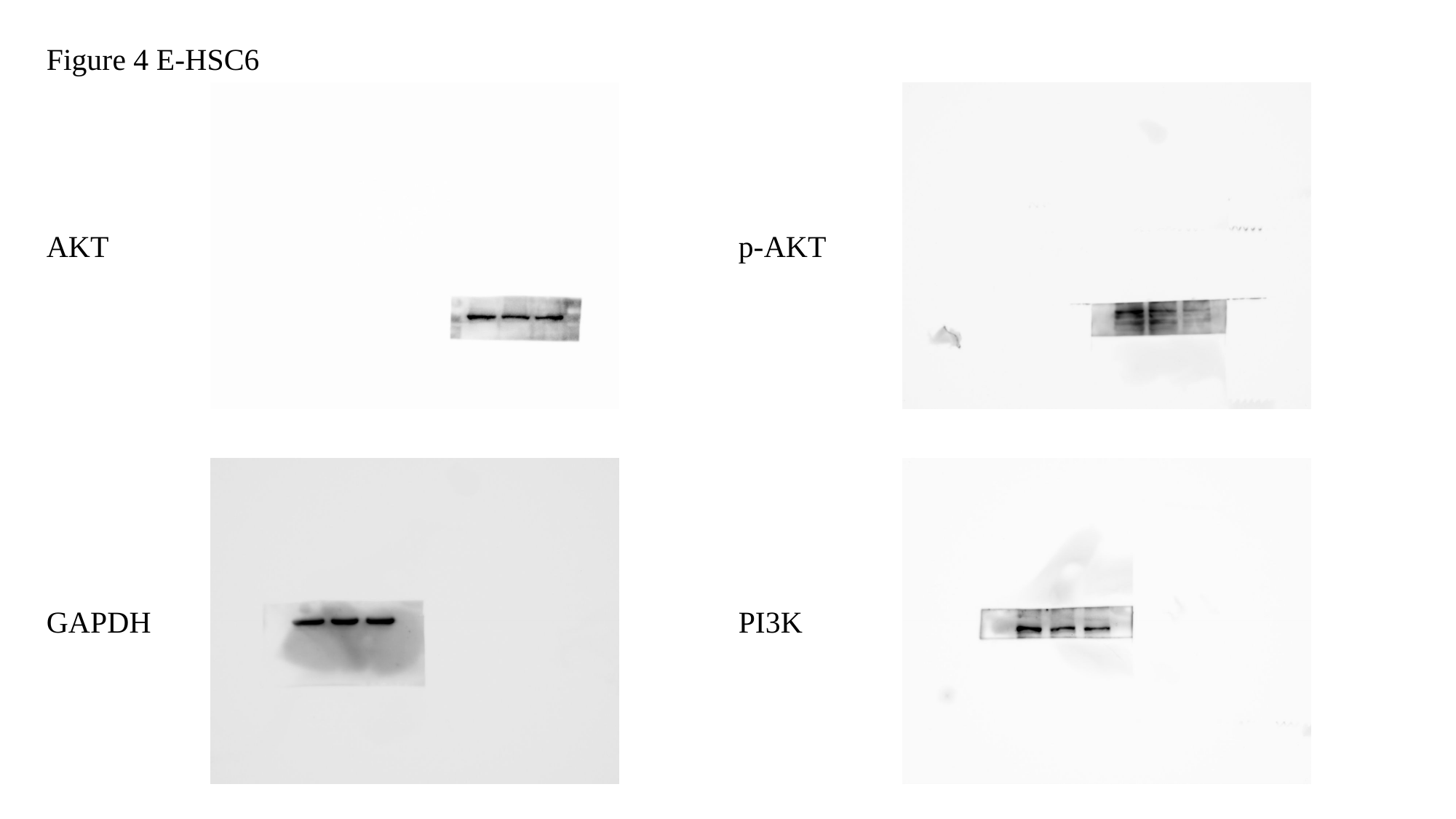

Figure 4 E-HSC6
AKT
p-AKT
GAPDH
PI3K

## Slide 18
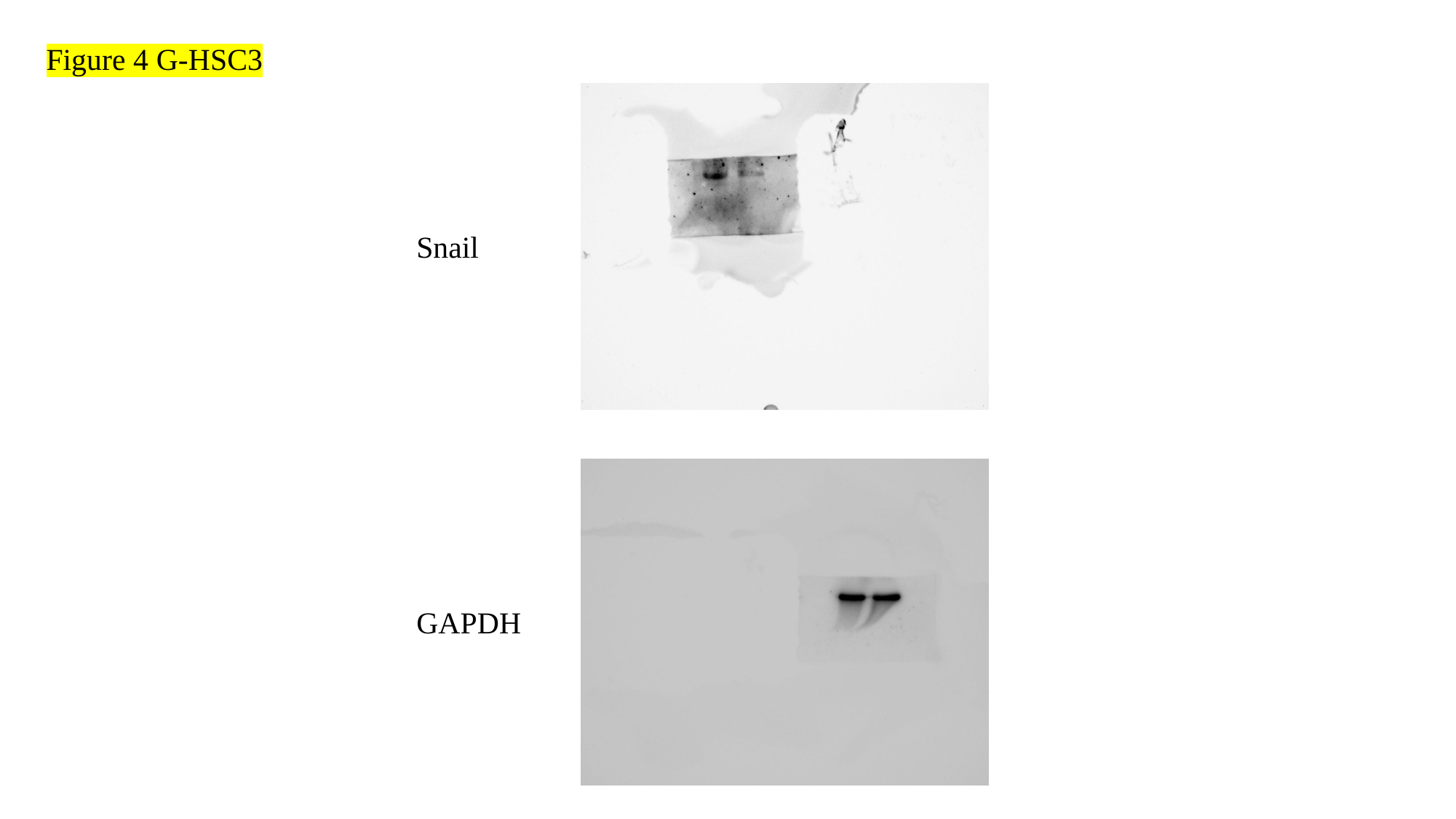

Figure 4 G-HSC3
Snail
GAPDH

## Slide 19
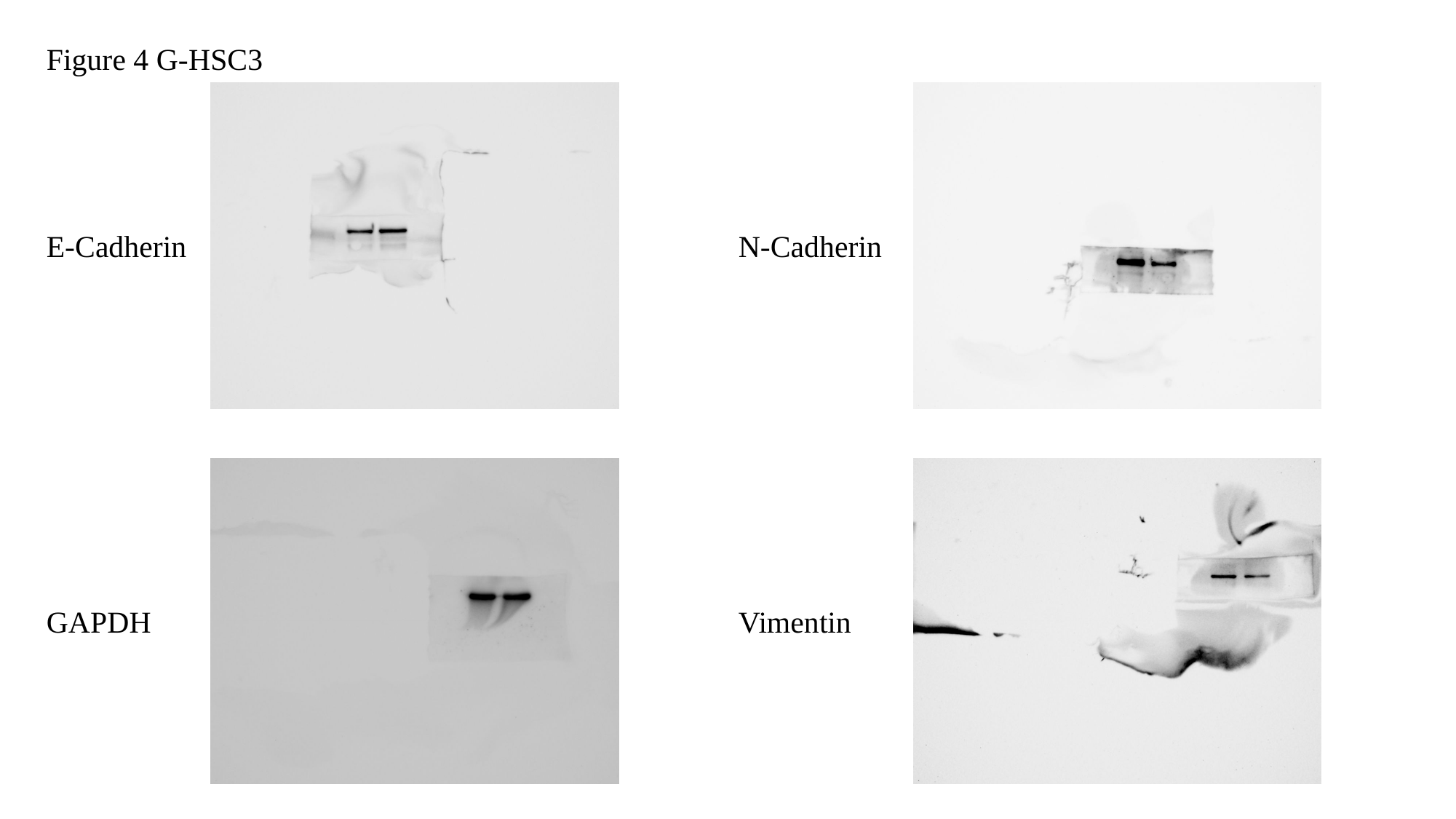

Figure 4 G-HSC3
E-Cadherin
N-Cadherin
GAPDH
Vimentin

## Slide 20
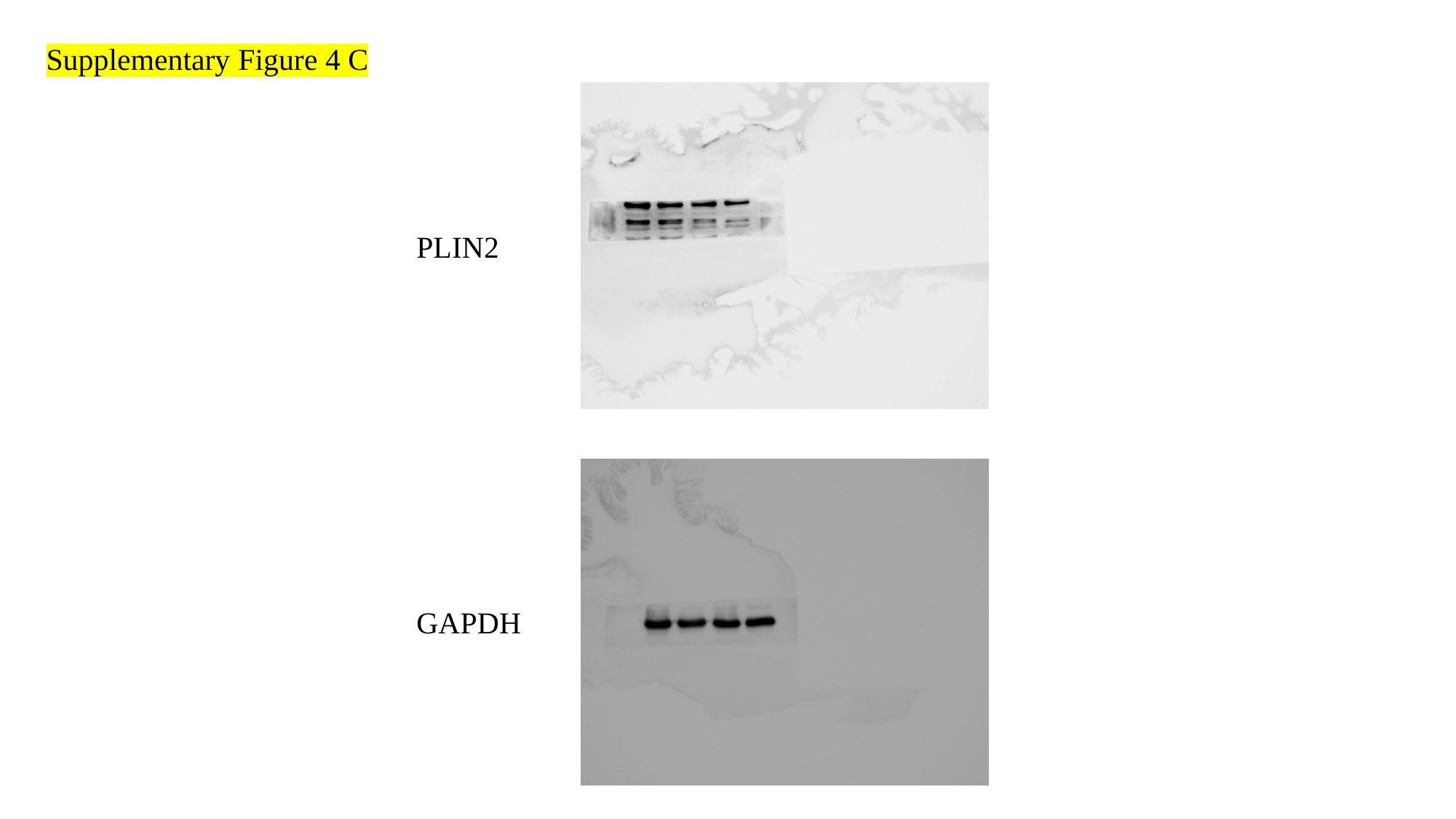

Supplementary Figure 4 C
PLIN2
GAPDH
